# Single-nuclei transcriptomes from human adrenal gland reveal distinct cellular identities of low and high-risk neuroblastoma tumors

**DOI:** 10.1038/s41467-021-24870-7

**Published:** 2021-09-07

**Authors:** O. C. Bedoya-Reina, W. Li, M. Arceo, M. Plescher, P. Bullova, H. Pui, M. Kaucka, P. Kharchenko, T. Martinsson, J. Holmberg, I. Adameyko, Q. Deng, C. Larsson, C. C. Juhlin, P. Kogner, S. Schlisio

**Affiliations:** 1 Department of Microbiology, Tumor and Cell Biology, Karolinska Institutet, Stockholm, Sweden; 2 Department of Physiology and Pharmacology, Karolinska Institutet, Stockholm, Sweden; 3 Max Planck Institute for Evolutionary Biology, Plön, Germany; 4 Department of Biomedical Informatics, Harvard Medical School, Boston, MA USA; 5 Harvard Stem Cell Institute, Cambridge, MA USA; 6 Department of Pathology and Genetics, University of Gothenburg, Sahlgrenska University Hospital, Gothenburg, Sweden; 7 Department of Cell and Molecular Biology, Karolinska Institutet, Stockholm, Sweden; 8 Department of Neuroimmunology, Center for Brain Research, Medical University of Vienna, Vienna, Austria; 9 Department of Oncology-Pathology, Karolinska Institutet, Stockholm, Sweden; 10 Women’s and Children’s Health, Karolinska Institutet, Stockholm, Sweden

**Keywords:** Cancer genetics, Paediatric cancer, Cancer genetics

## Abstract

Childhood neuroblastoma has a remarkable variability in outcome. Age at diagnosis is one of the most important prognostic factors, with children less than 1 year old having favorable outcomes. Here we study single-cell and single-nuclei transcriptomes of neuroblastoma with different clinical risk groups and stages, including healthy adrenal gland. We compare tumor cell populations with embryonic mouse sympatho-adrenal derivatives, and post-natal human adrenal gland. We provide evidence that low and high-risk neuroblastoma have different cell identities, representing two disease entities. Low-risk neuroblastoma presents a transcriptome that resembles sympatho- and chromaffin cells, whereas malignant cells enriched in high-risk neuroblastoma resembles a subtype of TRKB+ cholinergic progenitor population identified in human post-natal gland. Analyses of these populations reveal different gene expression programs for worst and better survival in correlation with age at diagnosis. Our findings reveal two cellular identities and a composition of human neuroblastoma tumors reflecting clinical heterogeneity and outcome.

## Abstract (web summary)

Childhood neuroblastoma can be separated into high and low risk groups, with prognosis depending on age at diagnosis. Here, the authors show that low and high risk neuroblastoma tumours are composed of different cell types with different malignancy potential.

## Introduction

Neuroblastoma (NB) is a pediatric cancer arising from the sympathoadrenal cell lineage frequently originating in the adrenal glands (AG)^1^. This malignancy represents 8–10% of all childhood cancer cases, and is responsible for 15% of all pediatric oncology deaths worldwide^2^. A clinical hallmark of neuroblastoma is heterogeneity, featuring outcomes ranging from lethal progression to spontaneous regression. The risk classification predicting the clinical behavior of the malignancy and its response to treatment, utilizes the INRGSS criteria (i.e., International Neuroblastoma Risk Grouping Staging System)^1,3^. One of the most significant and clinically relevant factors for this risk classification is age. Children younger than 18 months at the time of diagnosis display better prognosis (i.e., low-risk) than children diagnosed at a later age, and aging is in turn associated with poorer outcome (i.e., high-risk)^4,5^. Other prognostic markers are used to assign patients to specific risk groups, for example, ploidy, chromosomal alterations, *MYCN* amplification, and expression of neurotrophin receptors, such as TRKB (encoded by *NTRK2*) associated with high-risk and poor outcome. In contrast, neurotrophin receptor TRKA expression (encoded by *NTRK1)* is associated with low-risk and favorable outcome^2^. The reason why the age of the patient at the time of diagnosis is one of the strongest predictor of risk and outcome is not understood.

Previously, it has been reported that the majority of mouse chromaffin cells forming the adrenal medulla originate from an embryonic neural-crest progeny, specifically, from multipotent Schwann cell precursors (SCPs). SCPs are nerve associated cells that migrate along the visceral motor nerves to the vicinity of the developing adrenal gland, and form ~80% of the chromaffin cells^6^. The remaining 20% is directly derived from a migratory stream of neural-crest cells (NCCs) that commit to a common sympathoadrenal lineage in proximity to the dorsal aorta^7,8^, and is considered as the source of neuroblastoma^9,10^. SCPs also give rise to paraganglia during mouse embryonic development, such as chromaffin cells in Zuckerkandl’s organ (ZO) and to some sympathetic neurons^11^. In mouse, the ZO reaches a maximum cell number shortly before birth, in contrast to human where the peak of cell number is reached around the third year of life, indicating species-specific developmental differences^12^. However, SCPs are retained for a rather short time during mouse embryonic development and disappear around E15^6^. Thus, it remains unknown how human chromaffin cells are produced and regenerated after birth and if any distinct population of cells serves as their progenitor pool. We hypothesize that postnatal chromaffin cells are derived from a different source than their developmental origin (namely SCPs), and aberrations in this cell source could explain the age-dependent risk stratification of neuroblastoma.

Here we deep-sequence full-length coverage RNA from single nuclei of tumors (*n* = 11) across different risk groups and cross-compare cell clusters transcriptomes with healthy postnatal adrenal gland (human *n* = 3, mouse *n* = 5). We further include recently published single-cell sequencing datasets from 10X single-sequenced NB tumors (*n* = 8)^13^, E12–E13 embryonic mouse adrenal anlagen^6^, human fetal adrenal gland^14^, and the transcriptional profiles of neuroblastoma mesenchymal-/NCC-like and (nor-)adrenergic cell lineages^15,16^. We identify a cluster of TRKB+ cholinergic cells unique to human postnatal adrenal gland, that differ from previously described embryonic Schwann cell precursors (SCP). This TRKB+ population of cells shares a specific gene signature with a cluster of undifferentiated cells of mesenchymal nature enriched in high-risk neuroblastoma. The gene signature of these mesenchymal cells is in turn coupled with lower patient survival probability and older age-at-diagnosis when tested in a larger cohort of 498 neuroblastoma patients^17^. Conversely, more differentiated noradrenergic cells are over-represented in low-risk cases, and share specific gene signatures with adrenal human and mouse transcriptomes that resemble the signature of sympatho- and chromaffin cells. Our results suggest that high-risk neuroblastomas are characterized by a population of progenitor cells that resemble a cell type in postnatal adrenal gland with migratory and mesenchymal signatures, while the low-risk neuroblastoma resembles postnatal and developing chromaffin cells and sympathoblasts.

## Results

To understand why high-risk neuroblastomas arise in children older than 18 months, we first cataloged normal cell populations in postnatal adrenal gland which is a common location for this pediatric malignancy. We define the identity of normal cell populations in the adrenal gland of both postnatal human (*n* = 3, 1536 single nuclei) and mouse (*n* = 5, 1920 single whole-cells) by single- nuclei/cell RNA-sequencing (SmartSeq2, see the “Methods” section) to an average depth of 485,000- and 669,000 reads per nuclei/cell, respectively (Supplementary Fig. 1a, b, Supplementary Data 1). Technical and biological features of the transcription profile acquired from nuclei (human) or from whole cells (mouse) are summarized in Supplementary Fig. 1c.

### Postnatal human and mouse adrenal glands share cell populations but exhibit differences in chromaffin cells

**Fig. 1:**
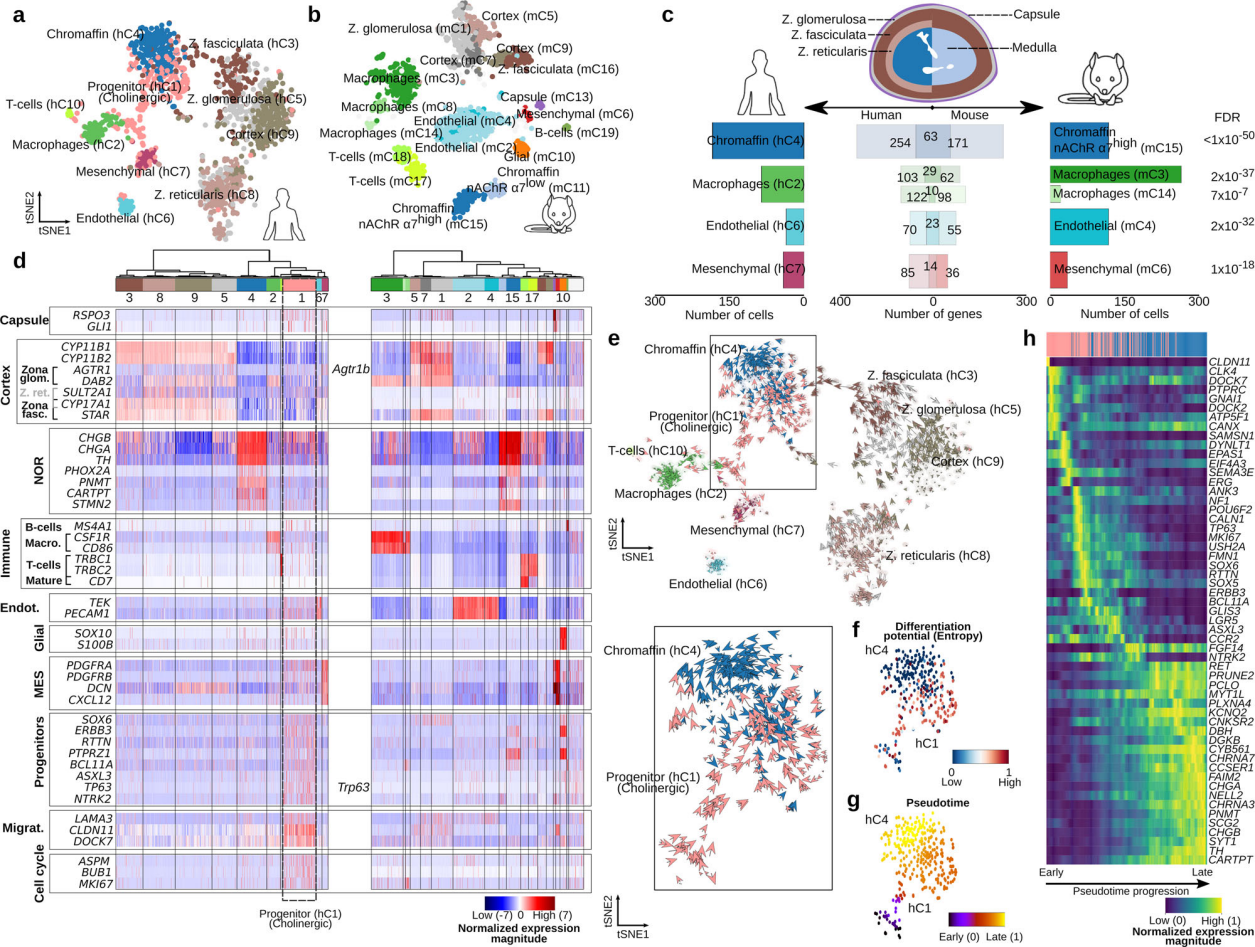
Anatomy of human and mouse adrenal gland (AG) revealed from single nuclei/cell analysis. **a** 1536 single nuclei of human AG from three different patients were sequenced with Smart-seq2 to an average depth of 485,000 reads per cell. Cells with high-quality (*n* = 1322) were selected and further processed with PAGODA. Cells were grouped into ten different clusters, including cortex (in brown and gray colors), chromaffin (blue hC4), mesenchymal (purple hC7), endothelial (light blue hC6), and immune cells (i.e., T cells hC10 and macrophages hC2 in green colors). **b** 1920 single cell of mouse AG from five different samples were sequenced with Smart-seq2 to an average depth of 670,000 reads per cell. Cells with high-quality (*n* = 1763) were selected and further processed with PAGODA. Cells were grouped into nineteen different clusters, including cortex (in brown and gray colors), chromaffin (blue mC15 and light blue mC11), mesenchymal (red mC6), capsule (purple mC13), endothelial (light blue mC2 and mC4), glial (orange mC10), and immune cells (i.e., T cells mC17 and mC18, and macrophages mC3, mC8, and mC14 in green colors). Human and mouse adrenal glands zonation^49^ is illustrated in (**c**). **c**, **d** A comparison of the specific gene signature between human and mouse revealed similar transcription signatures for mesenchymal, endothelial, and immune clusters, nevertheless, the two different postnatal chromaffin cells could only be differentiated in mouse. **d** Hierarchical clustering of cells and expression of a panel of markers including noradrenergic (NOR), mesenchymal (MES), endothelial (Endot.), and migratory (Migrat.). **e**–**g** A population of cells with a significant high expression of progenitor markers (i.e., *SOX6*, *ERBB3*, *RTTN*, FDR<0.01) and high differentiation potential found uniquely in human AG, is sourcing the chromaffin cells as indicated by velocity, entropy, and pseudotime analyses. **h** In this process, gene expression elapses from an undifferentiated stem-like- (i.e., *RTTN+*) to an adrenergic signature (*PNMT+*), passing by a noradrenergic stage (i.e., *DBH+*), as indicated by the pseudotime of the underlying cellular process. Results in (**c**), (**e**), and the top inserts in (**d**) and (**h**) represent cell clusters by color in (**a**).

**Fig. 2:**
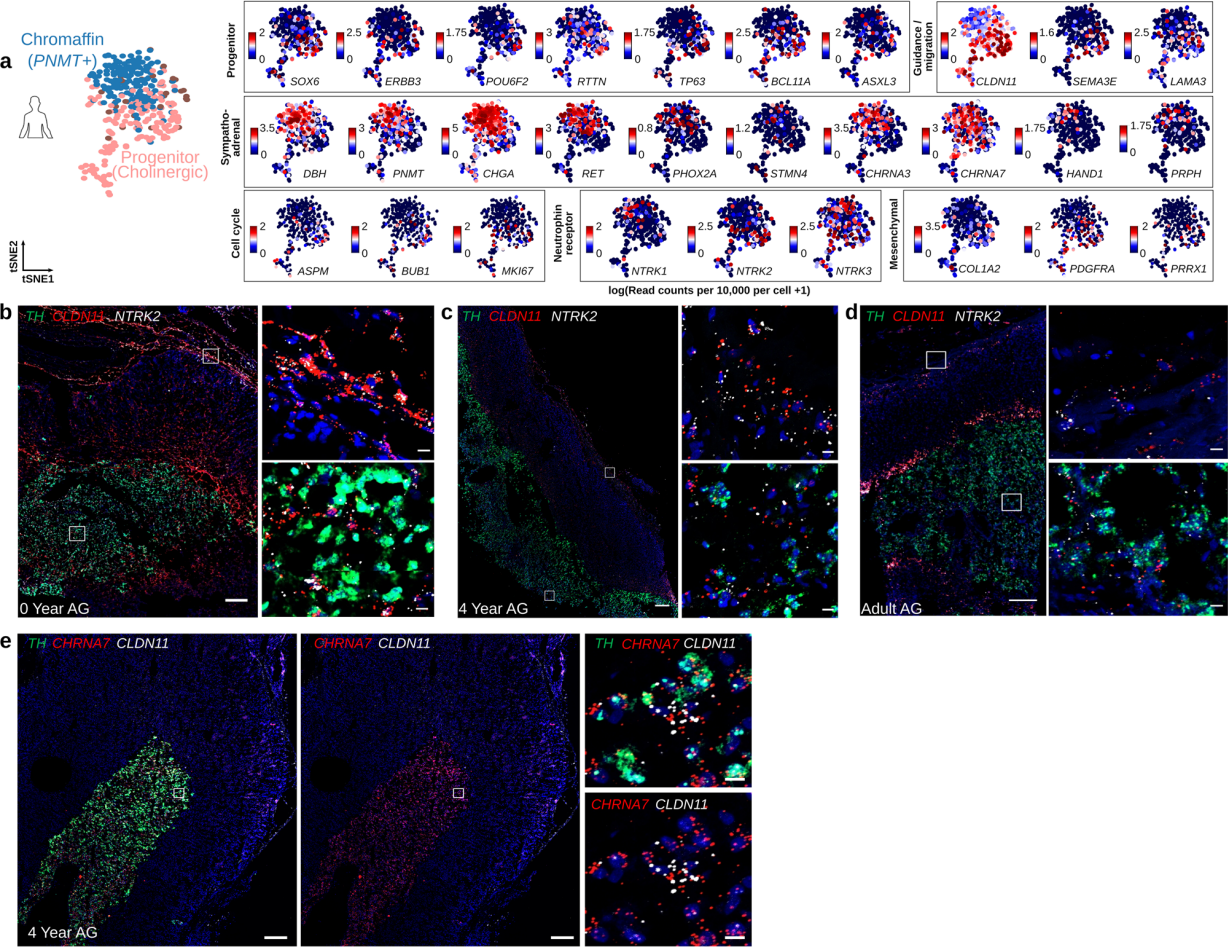
Location of human cholinergic progenitor (*NTRK2+ CLDN11+*) and chromaffin (*TH+*) cells within the postnatal human adrenal gland (AG). **a** tSNEs representing the expression of indicated genes in human cholinergic progenitor (pink) and chromaffin (blue) populations. The bars next to the tSNEs illustrate the expression measured as the logarithm of the read counts per 10,000. **b**–**e** Overview of tile-scanned images (×20) of postnatal human adrenal glands (AG) at indicated age. Scalebar of overview: 200 μm, zoom of boxed image: 10 μm. **b**–**d** RNAscope in situ hybridization (ISH) for *TH* (green)*, CLDN11* (red), and *NTRK2* (white) mRNA and counter stained with DAPI (blue)*. NTRK2*+ *CLDN11+* double positive cells were found in adrenal capsule and medulla exclusive from *TH* positive cells. **e** RNAscope ISH of a 4-year-old AG labeled with for *TH* (green)*, CHRNA7* (red), and *CLDN11* (white) mRNA and nuclear counter-stain (DAPI) as indicated. For all RNAscope experiments, the signal distribution patterns and cell morphological features were shown by the different combination of probes and independently reproduced three times on different samples.

The cell populations in human and mouse adrenal glands were annotated under the expectation of recovering both adrenal cortex- and medulla-associated cells (Fig. 1a for human, 1b for mouse, Supplementary Data 2). A reference guide of normal adrenal cell populations was generated by assigning an identity to each cluster, by cross-referencing significantly upregulated transcripts with canonical markers curated from the literature (Supplementary Data 2). Adrenal medulla cells were identified by the expression of a panel of nor- and adrenergic markers, including *PNMT*, *TH*, *DBH*, *CHGA*, and *CHGB* (Fig. 1d, Supplementary Data 2). In human, hC4 was identified as the chromaffin cell cluster (“NOR” panel were significantly upregulated, FDR < 0.01, Welch’s *t-*test, Fig. 1a, d, Supplementary Data 3, whereas in mouse two chromaffin cell clusters (i.e., mC11 and mC15) were identified (noradrenergic markers exemplified in “NOR” panel were significantly upregulated, FDR < 0.01, Welch’s *t-*test, Fig. 1b, d, Supplementary Data 2, Supplementary Data 4). Nevertheless, the mouse chromaffin population mC15 shared a more significant specific gene signature with the human chromaffin cluster hC4 (FDR < 0.01, Fisher’s exact test, Fig. 1c, Supplementary Data 5). To understand the differences between the two mouse chromaffin populations (mC11 and mC15), we investigated the expression of genes that differed significantly between them. The expression of *CHGA*, *CHGB*, *PHOX2A*, and *PHOX2B* were significantly higher in mC15 than in mC11 (FDR < 0.01, Welch’s *t-*test), while the expressions of *PNMT* was higher in mC11 than in mC15 (FDR < 0.01, Welch’s *t-*test, Fig. 1d, Supplementary Data 2). In addition, mC15 cluster exhibited a significantly higher expression of a different repertoire of cholinergic muscarinic and nicotinic receptors (mAChR and nAChR) than mC11 including *CHRM1*, *CHRNA3*, *CHRNA7*, and *CHRNB4* (FDR < 0.01, Welch’s *t-*test, Supplementary Fig. 2a, b, Supplementary Data 2). In contrast to mouse chromaffin cells, human postnatal *PNMT*+ chromaffin cells (cluster hC4) showed a significant expression of the sympathoblast marker *PRPH* (FDR < 0.01, Welch’s *t-*test, Fig. 2a).

### Identification of a cholinergic progenitor population in the human postnatal adrenal gland

Unexpectedly, we found a population of cells unique to the human postnatal adrenal gland (hC1) which significantly expressed various progenitor and migratory markers, including *SOX6, BCL11A, ERBB3, NTRK2* (encoding for TRKB)*, RTTN, PTPRZ1, TP63, ASXL3, POU6F2*, *SEMA3E*, *LAMA3*, *DOCK7*, and *CLDN11* (FDR < 0.01, Welch’s *t-*test, “Progenitors” and “Migrat.”, highlighted by a dotted line, Figs. 1d and 2a, Supplementary Data 2 and Supplementary Data 3). Furthermore, hC1 progenitor population presented a proliferating nature and significantly expressed cell cycling genes such as *MKI67, ASPM*, and *BUB1* (“Cell Cycle” panel was significantly upregulated FDR < 0.01, Welch’s *t-*test, Figs. 1d and 2a and Supplementary Data 3). These progenitors, however, did not express previously described multipotent Schwann cell precursors (SCPs) markers such as *SOX10*, *FOXD3*, and *S100B* (Fig. 1d, Supplementary Data 2). In support of this finding, previously described SCPs from mouse adrenal anlagen at embryonic day E12/E13^6^, and from human fetal adrenal glands at 8–14 post-conception weeks (PCW)^14^ shared no significant specific gene signature with the human postnatal progenitor cluster hC1 (Fisher’s exact test, Supplementary Fig. 2c, d, Supplementary Data 5). No progenitor cells other than SCP, chromaffin, and sympathoblast populations have been described for fetal adrenal gland^13,14^. In this regard, the only gene signatures shared with human fetal adrenal gland and mouse embryonic anlagen belonged to cell-cycle genes expressed in cycling sympathoblast (mouse E13 and human 8–14 PCW) and cycling chromaffin (human 8–14 PCW, FDR *<* 0.05, Fisher’s exact test, Supplementary Data 5). An estimation of cell velocities (i.e., computational reconstructions of cells trajectory and fates that uses transcript splicing to calculate the direction and speed of differentiation)^18,19^ suggest that this type of cells (i.e., hC1) repopulates chromaffin cells in postnatal human adrenal gland (Fig. 1e). A velocity-driven gene trajectory analysis using pseudotime indicates that the progenitor cells transit from precursors cells with high differentiation potential to differentiated chromaffin cells (Fig. 1f–h). Furthermore, both progenitor (hC1) and chromaffin population (hC4), express the nicotinic acetylcholine receptor nAChRα7 (*CHRNA7*), suggesting that progenitor cells are cholinergic in nature (FDR *<* 0.01, Welch’s *t-*test, Supplementary Data 2).

To validate the expression and spatial context of the human cholinergic progenitor cells (hC1), a series of RNAscope in situ hybridizations (ISH) was performed in postnatal adrenal glands from 0- and 4-year-old children and adults (Fig. 2b–e, Supplementary Figs. 2e and 3). We first elucidated the anatomy of the glands in each section for medulla (*TH*), cortex (*CYP11B2)*, and capsule *(RSPO3)* (Supplementary Fig. 2e). To identify cells belonging to the hC1 progenitor population, we tested markers identified as significantly upregulated in hC1 population: *NTRK2* and *CLDN11* (Fig. 2b–d and Supplementary Fig. 3a–c). *NTRK2+CLDN11+* double positive cells were found in human adrenal capsule and medulla exclusive from *TH+* cells at all ages, but most abundantly at 0 year of age, suggesting that these cells decline over age. To confirm that the postnatal progenitor *CLDN11+* cells are of cholinergic nature, we performed in situ RNA-hybridizations with the cholinergic nicotinic receptor *CHRNA7* (i.e., nAChR α7) together with *TH* and *CLDN11*. Both cell types, *TH+* chromaffin and *CLDN11+* progenitor cells, express nAChR α7 (Fig. 2e). To further confirm that the postnatal progenitor cells in human postnatal gland are different from the previously described embryonic multipotent SCPs, we performed in situ RNA-hybridizations together with SCP/glia marker *SOX10* (Supplementary Fig. 3d–f). *NTRK2+* cells were exclusive from *SOX10+* cells in human postnatal adrenal gland at all ages.

### Different neuroblastoma risk groups present differences in cell population composition

**Fig. 3:**
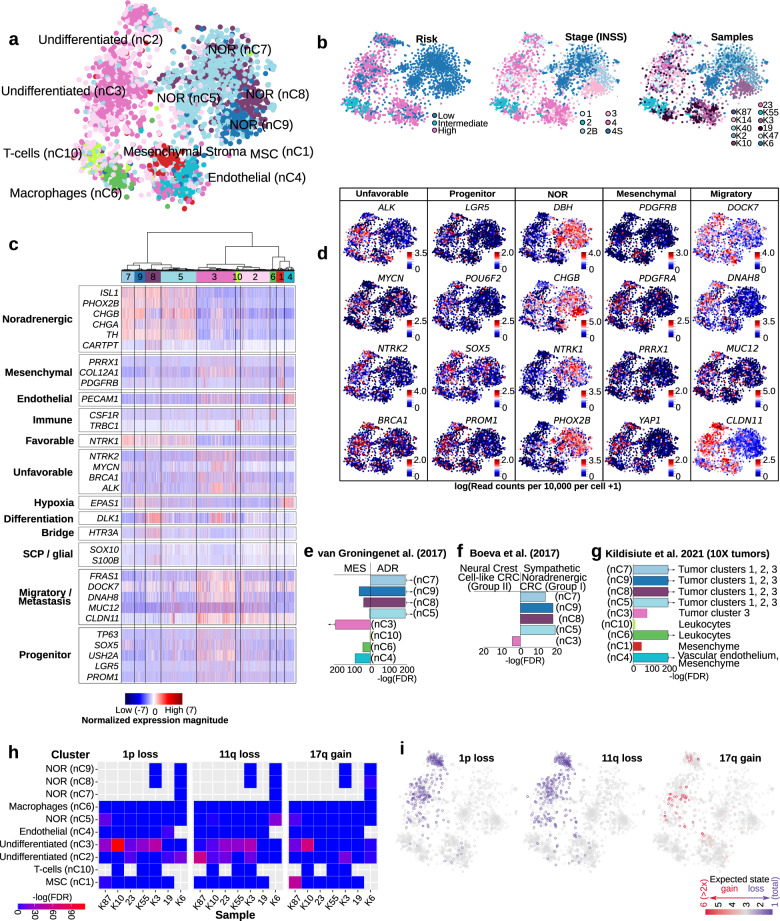
Intratumoral heterogeneity of childhood neuroblastoma (NB). 4224 single nuclei of human NB tumors were sequenced to an average depth of 676,000 reads per cell. **a** Cells with high-quality (*n* = 3212) were grouped into ten different clusters with PAGODA. NB risk groups, INSS stages, and samples are exemplified in (**b**). **c** Representation of the hierarchical clustering of cells: undifferentiated (light pink nC2 and pink nC3), mesenchymal stroma (MSC red nC1), endothelial (light blue nC4), T-cells (light green nC10), macrophages (green nC6), and noradrenergic (“NOR”, blue nC5, nC7, nC8, and nC9) and various markers of interest (also in insert **d**). The bars next to the tSNEs in (**d**) illustrate the expression measured as the logarithm of the read counts per 10,000. **e**, **f** Using a gene enrichment-based approach, the noradrenergic clusters of NB (nC5, nC7, nC8, and nC9) proved to share a significant number of upregulated genes with sympathetic noradrenergic^15^ and “ADR” (i.e., adrenergic)^16^ transcriptional signatures. Oppositely, the undifferentiated cluster nC3 shared a significant number of upregulated genes with neural-crest cell-like signature^15^ and MES (i.e., mesenchymal)^16^ signatures. **g** A comparison of the gene-specific signature of NB clusters with markers defining clusters in eight tumors previously sequenced with 10X^13^, suggests a more significant similarity of the noradrenergic clusters (nC5, nC7, nC8, and nC9) with tumor clusters 1, 2, and 3; and of the undifferentiated nC3 cluster with the tumor cluster 3^13^. Only clusters with the most significant similarities are displayed. **h** An analysis of the CNVs indicates a significant number of cells with rearrangements (indicated by red, Fisher exact tests) in the undifferentiated cluster nC3 for most samples, while a remarkable number of rearrangements was observed in fewer samples for the NOR (nC5), MSC (nC1), and undifferentiated nC2 clusters. **i** A subset of the predicted CNVs was validated for each sample, and illustrated in the tSNE for cells with an expected rearrangement (i.e., expected state) overlapping the validated region. Colors displayed in (**e**), (**f**), and (**g**) represent cell clusters by color in (**a**). An arrow next to the bar indicates a FDR < 1 × 10^−50^.

Next, we used single-nuclei transcriptomics to characterize eleven neuroblastoma samples across different clinical risk groups, and genetic subsets (Fig. 3a, b, Supplementary Data 1). Deep-frozen samples obtained from surgical resections were used for nuc-Seq (SmartSeq2), yielding a total of 3212 high-quality nuclei with an average of 709,676 high-quality reads per nuclei (see “Methods” and Supplementary Fig. 1). Cluster analysis using PAGODA identified ten cell populations, classified as undifferentiated (nC2 and nC3), noradrenergic (NOR clusters nC5, nC7, nC8, and nC9), and stroma clusters: mesenchymal stroma (MSC nC1), endothelial (nC4), macrophages (nC6), and T cells (nC10) (Fig. 3a, Supplementary Data 6). The identity of each cluster was assigned by cross-referencing cluster-defining transcripts with canonical markers curated from the literature (Supplementary Data 2). Clusters nC2 and nC3 (referred as “undifferentiated”), presented a significant high expression of progenitor markers *PROM1, RTTN, ERBB3, POU6F2*, and the migratory marker *CLDN11*. Remarkably, the undifferentiated nC3 clusters presented a significantly high expression of *MYCN, ALK, BRCA1*, and *BRCA2* genes and progenitor markers *BCL11A, NTRK2, SOX5, SOX6, TP63, LGR5,* and *USH2A* (FDR < 0.01, Welch’s *t*-test, Fig. 3c and Supplementary Data 2, Supplementary Data 6). Oppositely, the noradrenergic clusters (NOR, nC5, nC7, nC8, and nC9) expressed significantly high *NTRK1* (TRKA) and noradrenergic markers *TH, DBH, PHOX2A, PHOX2B*, and *ISL1* (FDR < 0.01, Welch’s *t-*test, Fig. 3c, d and Supplementary Data 2, Supplementary Data 6).

Neuroblastoma samples of different clinical risk groups and stages contained different contribution of cells within these clusters reflecting the clinical heterogeneity of these clinical groups. High-risk cases had a significantly higher proportion of cells in stroma and undifferentiated clusters, whereas the low-risk ones including spontaneous regressing (4S) cases, consisted mostly of cells belonging to the noradrenergic (NOR) clusters (FDR < 0.01, Chi-square test, Fig. 3b, Supplementary Fig. 4). In agreement with these observations, a deconvolution of 172 neuroblastoma bulk-sequenced samples (NB172 NCI TARGET project) indicated that a significantly higher proportion of cells in the stroma and undifferentiated nC3 clusters was present in high-risk patients (*n* = 139), and a higher proportion of cells in the noradrenergic clusters nC7, nC8, and nC9 in low-risk cases (*n* = 14, FDR < 0.01, Chi-square test, Supplementary Fig. 4). Some differences observed between single-nuclei and bulk-seq for nC2 and nC5 clusters could be a consequence of the clinical heterogeneity within patients of the same risk groups (Supplementary Fig. 4).

Mesenchymal markers *PRRX1*, *YAP1*, and *PDGFRA* were significantly expressed in both clusters, undifferentiated nC3 and MSC nC1 clusters (FDR < 0.01, Welch’s *t-*test, Fig. 3c, d and Supplementary Data 2), in contrast to *PDGFRB* whose upregulation was significantly higher in MSC cells (nC1, FDR < 0.01, Welch’s *t-*test, Fig. 3c, d and Supplementary Data 2). Consistently with this observation, the undifferentiated nC3 cluster shared a significant number of upregulated genes with the neural-crest cells-like signature (group II) and the mesenchymal (MES) gene expression signatures previously described in neuroblastoma cell lines^15,16^, while the noradrenergic clusters nC5, nC7, nC8, and nC9 presented a significantly high expression of genes characterizing the sympathetic noradrenergic (group I) and ADR (adrenergic) signatures (FDR < 0.01, Fisher’s exact test, Fig. 3e, f, Supplementary Data 7). In addition, the specific gene signatures from each neuroblastoma cluster were compared with the recently reported markers for six cell clusters in eight GOSH-cohort tumors sequenced with 10X^13^. The noradrenergic clusters nC5, nC7, nC8, and nC9 resembled more significantly the GOSH sympathoblast-like tumors clusters 1, 2, and 3, whereas the undifferentiated cluster nC3 showed a higher significant resemblance with the less differentiated GOSH-tumor cluster 3 (FDR = 2.87 × 10^−36^, Fisher’s exact test, Fig. 3g, Supplementary Data 7).

To differentiate malignant- from stroma cell clusters, an analysis of the genome rearrangements was conducted, and further experimentally validated. Significant copy number changes were computed for each sample using as background the changes observed in immune cells (i.e., T cells nC10 and macrophages nC6, only in the seven samples with immune cells as detailed in “Methods”). For most of samples, a significantly higher number of cells presented gains in 17q, losses in 1p or in 11q, in the undifferentiated nC3 cluster (K87, K10, 23, K55, and K3). Fewer samples presented a remarkable number of cells with rearrangements in the MSC nC1 (K87), undifferentiated nC2 (23, K3, K6, and K87), and NOR nC5 clusters (K6 and K87, FDR < 0.05, Fisher’s exact test, Fig. 3h). 1p deletion was confirmed by microarrays for samples 23, K10, and K55, 11q loss for samples 23 and K87, and 17q gains for samples K10, K3, and K87. Cells with predicted significant gains and losses from these samples are illustrated in Fig. 3i.

**Fig. 4:**
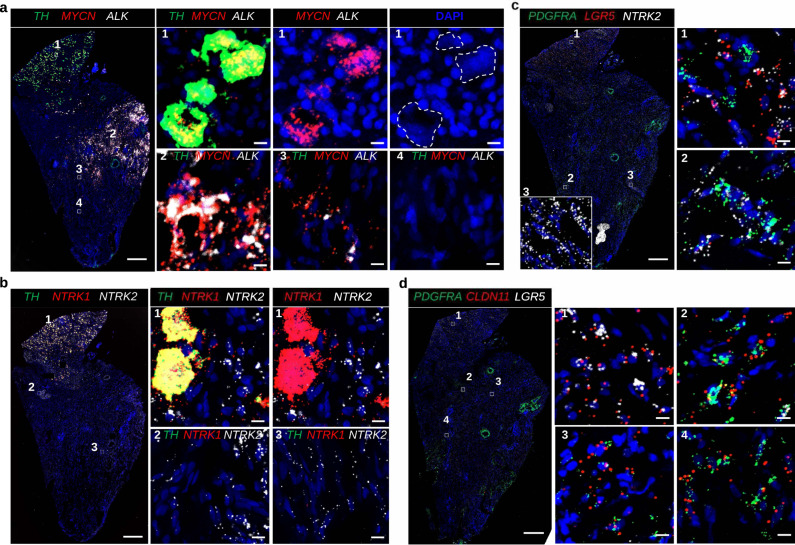
RNA in situ hybridization validating intratumoral heterogeneity in high-risk neuroblastoma stage 4. Overview of tile-scanned images (20x) high-risk neuroblastoma (K10, *MYCN* amplified) using RNAscope in situ hybridization. Scalebar of overview: 500 μm; zoom of boxed image: 10 μm. **a** Tumor labeled with RNAscope ISH for *TH* (green), *MYCN* (red), and *ALK* (white) mRNA, and counter stained with DAPI (blue). Dashed circles indicate cells with large nuclei in a region (box #1), that are *TH* and *MYCN* positive, but negative for *ALK*. Box #2 indicates part of the tumor with majority of cells double positive for *MYCN* and *ALK* but negative for *TH*. Box #4 indicates cells negative for all probes: *TH*, *MYCN*, and *ALK*. **b** Adjacent section from (**a**) labeled for *TH* (green), *NTRK1* (red), and *NTRK2* (white). Box #1 indicate cells with large nuclei in a region that are *TH+* and *NTRK1+*, and negative for *NTRK2*. Surrounding cells with small nuclei are positive for *NTRK2* only. Box #2,3 visualizes tumor region with majority of cells positive for *NTRK2* that are negative for *TH* and *NTRK1*. **c** Adjacent section stained for *PDGFRA* (green), *LGR5* (red), and *NTRK2* (white) mRNA is highlighting cells double positive for *LGR5* and *NTRK2* (box #1) or double positive for *PDGRFA*, *NTRK2* (box #2). Box #3 indicates a region of the tumor that is positive for *NTRK2* only. **d** Adjacent section stained for mesenchymal markers *PDGFRA* (green), *CLDN11* (red), and *LGR5* (white). Similar to **c**: some tumor regions (box #1) highlight cells double positive for *CLDN11* (red) and *LGR5* (white) that are negative for *PDGFRA* (box #1), as were other regions show cells that are double positive for *PDGFRA* (green) and *CLDN11* (red) (box #2, 3, and 4). For all RNAscope experiments, the signal distribution patterns and cell morphological features were shown by the different combination of probes and independently reproduced three times on different samples in (**a**) and (**b**), and independently reproduced four times on different samples in (**c**) and (**d**).

Next, we validated the expression of significantly enriched markers identified in neuroblastoma clusters (Fig. 4, Supplementary Fig. 5). RNAscope in situ hybridizations in a *MYCN*-amplified neuroblastoma case (K10, Supplementary Data 1) as independently confirmed by comparative genomic hybridization (CGH)^20^, revealed a high intratumoral heterogeneity for *TH*, *MYCN*, and *ALK* expression, with tumor regions with intense staining of *MYCN* and *ALK*, negative for *TH* (Fig. 4a, zoom 2). Other tumor regions revealed some diffuse stainings of few *TH+* positive cells with enlarged nuclei positive for *MYCN*, but negative for *ALK* (Fig. 4a, zoom 1), whereas other regions did not exhibit expression of *TH*, *MYCN*, and *ALK* (zoom 4). Interestingly, in situ hybridizations for *NTRK1* and *NTRK2* (encoding TRKA and TRKB, respectively) revealed that the identified *TH+* cells with enlarged nuclei are *NTRK1+* but *NTRK2*−, in contrast to all surrounding *NTRK1*− *NTRK2+* cells with small nuclei (Fig. 4b). It is possible that the identified *TH+* cells with enlarged nuclei (*TH+, MYCN+, NTRK1+, NTRK2*−*, ALK*−) might account for cells undergoing differentiation^21^. Expression of neurotrophin receptor *NTRK1* is characterized as a marker for favorable outcome and low-risk, while *NTRK2* expression is associated with poor outcome and high-risk^2^. In agreement with these findings, cells for the favorable low-risk neuroblastoma case (K6, stage 4S, Supplementary Data 1) were homogeneously *NTRK1+, TH+, NTRK2*− (Supplementary Fig. 5a).

In addition, we validated the expression of the mesenchymal marker *PDGFRA* that was identified to be significantly highly expressed in high-risk neuroblastoma cluster (nC3). *PDGFRA* expression in the high-risk *MYCN*-amplified case (i.e., K10) was observed in both *NTRK2+* (Fig. 4c zoom 2), and also in *CLDN11*+ cells (Fig. 4d, zoom 2, 3, and 4), whereas in *LGR5+* cells showed no *PDGFRA* signal (Fig. 4c zoom 1 and Fig. 4d zoom 1). In contrast, the low-risk stage 4S case was entirely negative for *PDGFRA* and *PRRX1* expression, however, homogeneously expressed in all cells noradrenergic markers *DBH* and *PHOX2B* (Supplementary Fig. 5b, c). Stage 2B neuroblastoma was more heterogeneous in *DBH* and *PHOX2B* expression (Supplementary Fig. 5d), with all cells lacking expression of *PRRX1* and very few cells positive for *PDGFRA* (Supplementary Fig. 5e).

### A cell population enriched in high-risk neuroblastoma resembles the human postnatal adrenal progenitor population

**Fig. 5:**
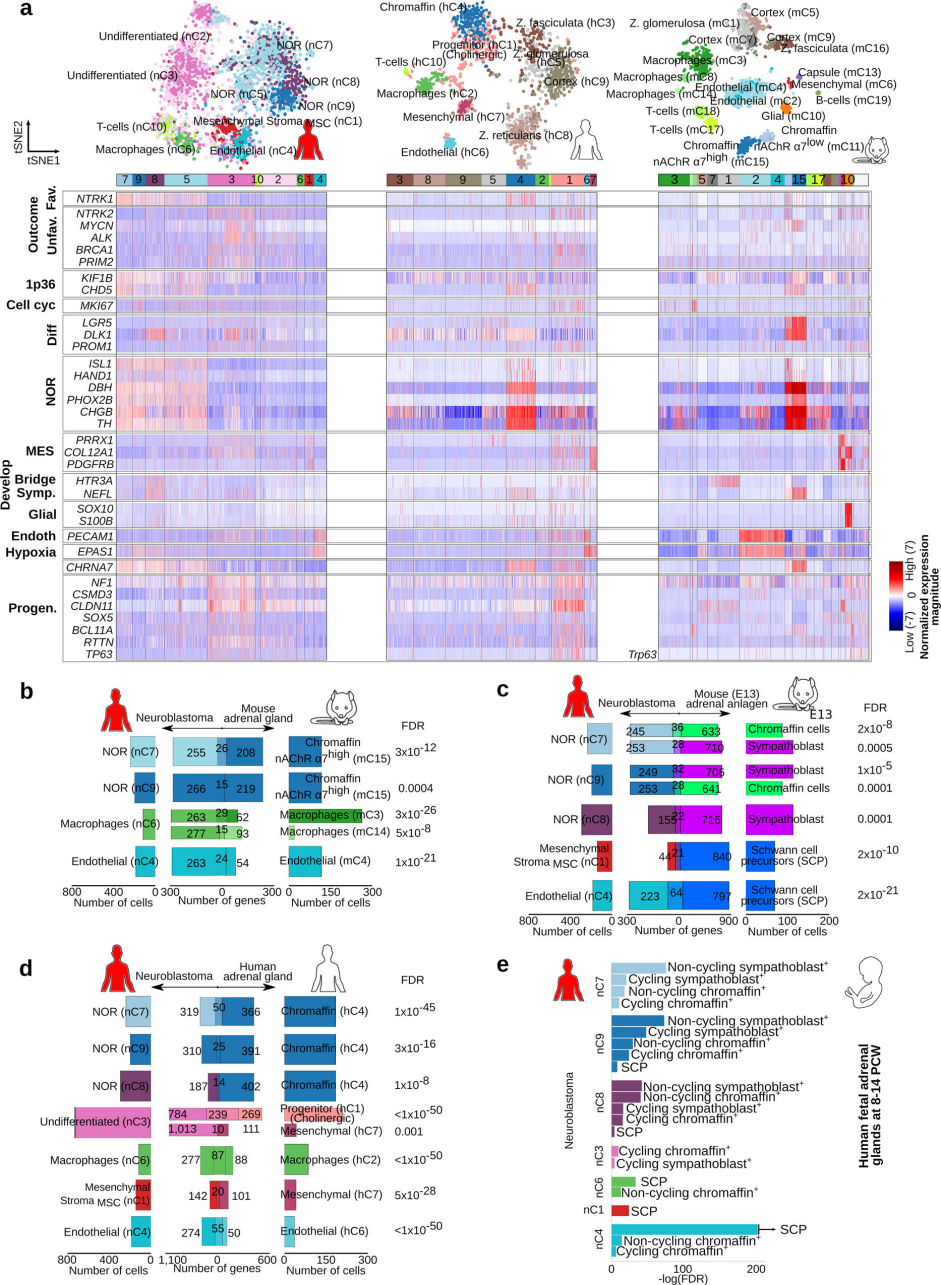
Cell clusters from neuroblastoma and healthy postnatal human and mouse adrenal glands share expression signatures. **a** Heatmap illustrating the normalized expression magnitude for selected genes organized following the hierarchical clustering (PAGODA) as shown in Figs. 1 and 3. **b**–**d** Venn diagrams illustrating the significantly shared specific gene signature. Shared genes are listed in Supplementary Data 8. Indicated FDRs were calculated with a Benjamini–Hochberg correction on *p*-values obtained with Welch’s *t*-tests as detailed in the “Methods”. **b** Venn diagrams of human neuroblastoma clusters compared to adult mouse postnatal clusters, **c** embryonic mouse clusters previously described^6^, and **d** human postnatal adrenal gland clusters. **e** Comparison of the specific signature from the human neuroblastoma clusters and the reported transcriptional signal for human fetal adrenal glands cell clusters (Dong et al.^14^). An arrow next to the bar indicates a FDR < 1 × 10^−50^. ^+^The original cluster annotations are currently debated and the included labels here correspond to those given by Kildisiute et al.^45^, and Bedoya-Reina and Schlisio^46^. Colors displayed in (**b**), (**c**), (**d**), and (**e**) represent cell clusters by color in (**a**).

**Fig. 6:**
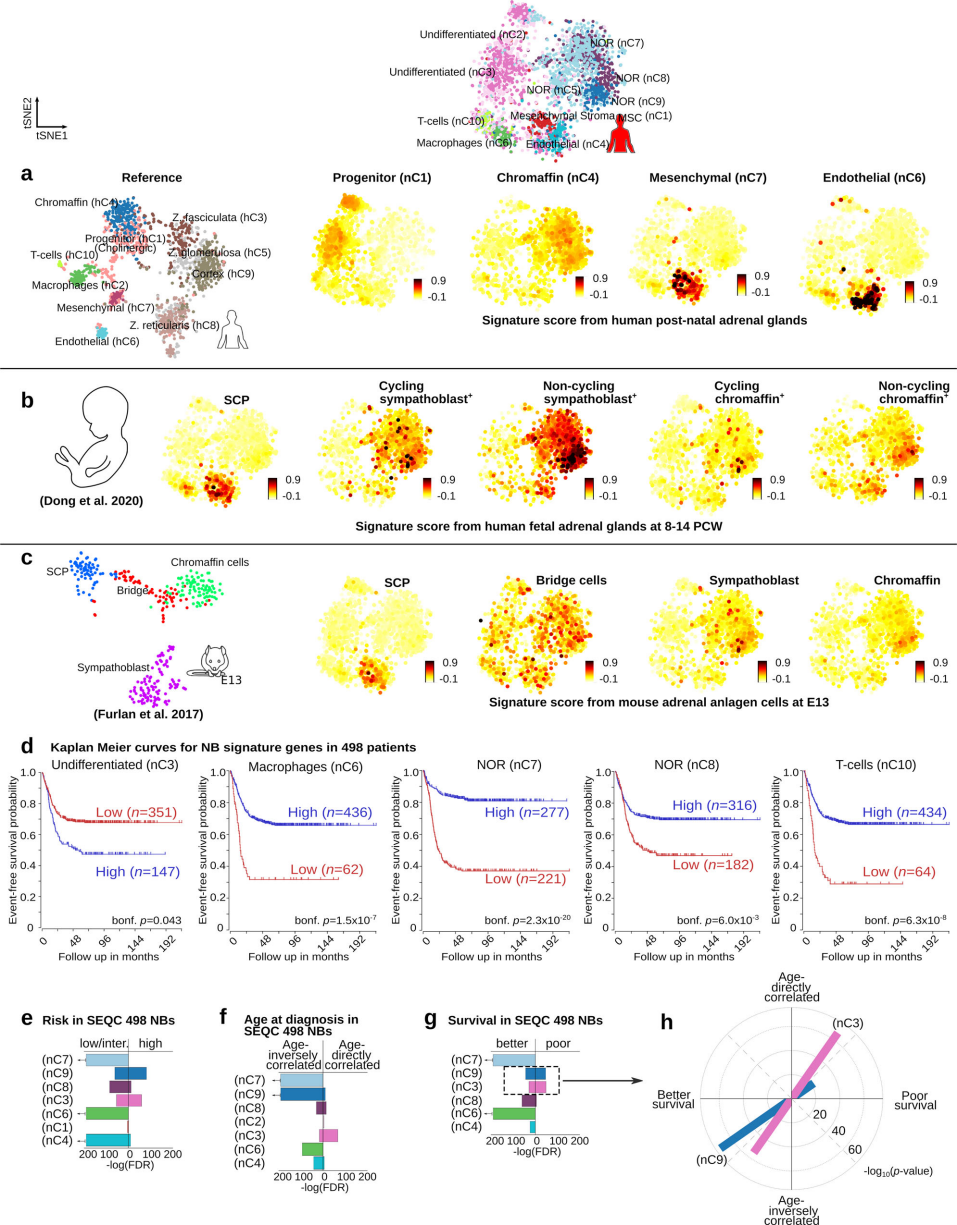
Cell clusters from neuroblastoma and developing human and mouse adrenal glands share distinct expression signatures, and their expression is associated with patient survival and age at diagnosis. tSNE of neuroblastoma clusters illustrating the signature score of genes (as detailed in “Methods”) characterizing **a** human postnatal, **b** embryonic^14^ adrenal glands, and **c** mouse embryonic adrenal anlagen (E13) cell clusters^6^. The bars next to the tSNEs in **a**, **b**, and **c** illustrate the scale of the signature score. A greater signature score indicates a larger transcriptional resemblance to the cluster of interest. SCP refers to the cluster of mouse multipotent Schwann cell precursors at E13; and Bridge refers to the cell cluster that defines transiting cells from SCP toward the chromaffin population at E13. **d** Kaplan–Meier curves for neuroblastoma clusters with significant differences (Bonferroni-corrected [bonf. *p*], log-rank tests in survival for 498 SEQC neuroblastoma patients with a low (red) and high (blue) signature gene expression^17^. **e**–**g** Using a gene enrichment-based approach (Benjamini–Hochberg corrected, Fisher’s exact tests), the specific signature genes for the noradrenergic nC7, nC8, and nC9, endothelial nC4, macrophages nC6, and undifferentiated nC3 clusters were found to be significantly upregulated in low- and intermediate (inter.)-risk patients and also in individuals with better survival. In contrast, the signature genes of noradrenergic nC9 and undifferentiated nC3 clusters presented a significantly enrichment in patients with high-risk and poor survival. The signature genes of the undifferentiated nC3 cluster presented also a remarkable significant correlation with age at diagnosis. **h** Signature genes associated with poor survival for noradrenergic nC9 and undifferentiated nC3 clusters are significantly correlated with age at diagnosis, and oppositely those associated with better survival are inversely correlated with age. Genes are included in Supplementary Data 9. A solid arrow next to the bar indicates a FDR < 1 × 10^−50^. Colors of bars displayed in (**e**), (**f**), (**g**), and (**h**) represent the neuroblastoma cell clusters by color in (**a**).

**Fig. 7:**
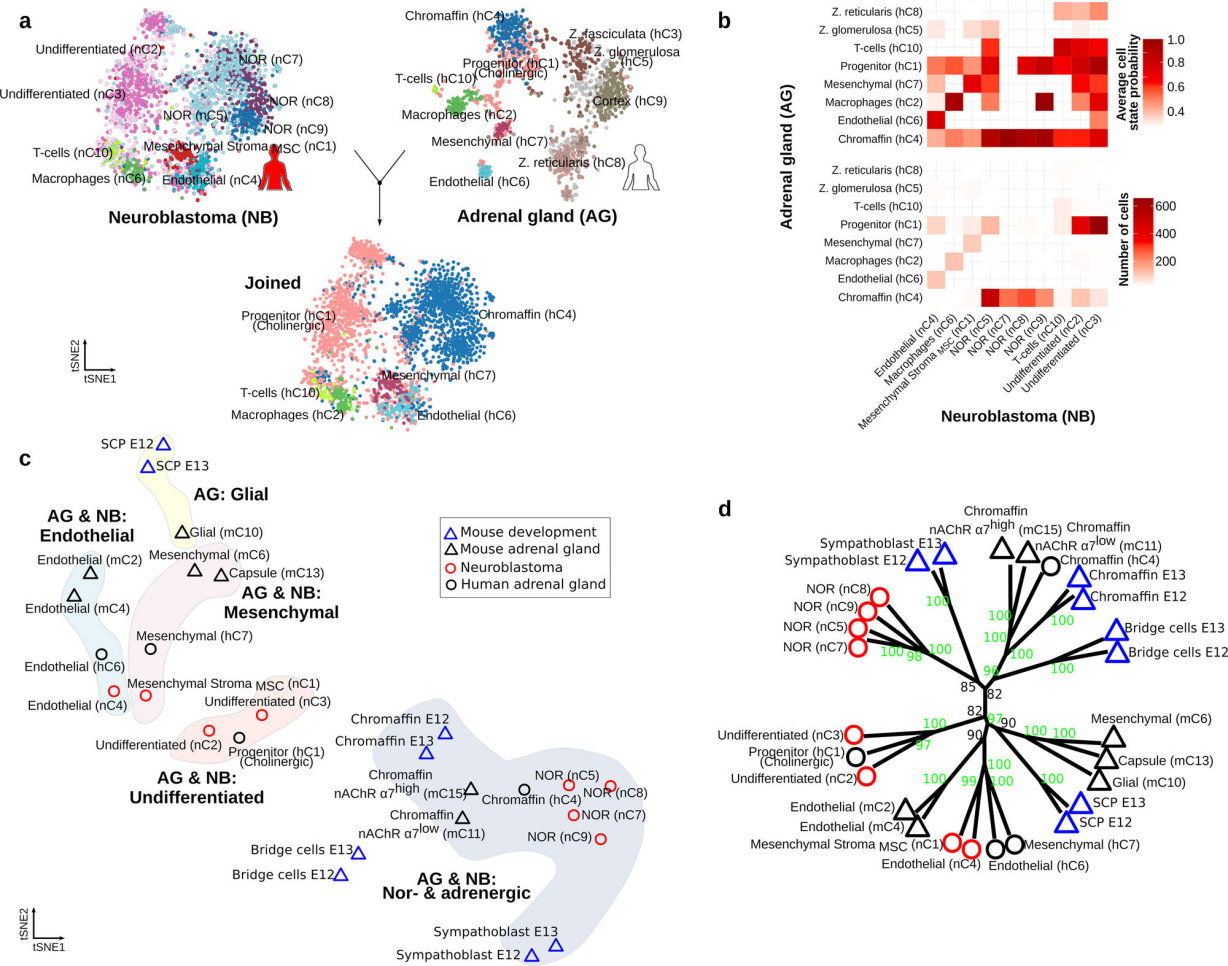
Commonalities in the transcriptional profile of cell populations in postnatal and developing human and mouse adrenal glands, and neuroblastoma. **a** tSNE illustrating the projection of postnatal human adrenal gland cell clusters (i.e., states, reference top right) onto neuroblastoma (query top left), based on the detection of mutual nearest neighbor cell anchors (as detailed in “Methods”). Joint visualization of the neuroblastoma cell clustering (bottom) after cell classification with colors corresponding to the transferred adrenal gland cell states. **b** Heatmaps of the average probabilities of best matching reference states for each query cluster (i.e., average cell state probability, top), including number of cells (bottom). **c** tSNE using Euclidean distances in the quartile-normalized matrix of the average normalized expression from single-cell/nuclei sequenced cell populations from (A) human (i) neuroblastoma and (ii) adrenal gland, and (B) mouse (i) adrenal gland and (ii) derived adrenal anlagen at E12 and E13^6^. Mesenchymal, endothelial, and neural-crest derived populations from human and mouse, adrenal gland, and neuroblastoma group accordingly to gene expression in (i) adrenergic and noradrenergic cells, (ii) undifferentiated cells, (iii) mesenchymal, and (iv) endothelial cells. **d** Hierarchical clustering of transcriptional similarities between cell clusters supported with unbiased *p*-values^41^. Green numbers in the branches indicate a high support (i.e., unbiased *p*-value > 95) that has a lowest support at 0 and a highest at 100.

High-risk neuroblastoma commonly arises in children older than 18 months and frequently originates in the adrenal glands^1^. To understand how the observed neuroblastoma populations in high and low-risk cases are related to normal postnatal and embryonic adrenal tissues, we performed a comparative analysis of their transcriptional profiles (Figs. 5, 6, and 7). Significant expression of (nor-)adrenergic (“NOR”) markers (i.e., *CHGA, CHGB, DBH, TH, PHOX2A,* and *PHOX2B*) is common for healthy sympatho-adrenal cells and tumor NOR populations identified in low-risk neuroblastoma cases (FDR < 0.01, Welch’s *t*-test, Fig. 5a, Supplementary Data 2). In agreement with this observation, specific gene signatures from neuroblastoma NOR clusters were found to be significantly shared with both, mouse embryonic (E12–E13) and human (8–14 PCW) chromaffin and sympathoblast clusters, and also with human and mouse postnatal chromaffin clusters (FDR < 0.01, Fisher’s exact test, Fig. 5b–e, Supplementary Data 8, Supplementary Fig. 6a). A more significant resemblance was observed between the NOR nC7 and nC9 clusters with fetal (8–14 PCW) sympathoblast cells (FDR = 4.47 × 10^−33^ and FDR = 5.38 × 10^−32^, respectively, Fisher’s exact test), while the NOR nC8 cluster presented a closer similitude to fetal (8–14 PCW) chromaffin and sympathoblast clusters (FDR = 5.45 × 10^−18^ and FDR = 1.08 × 10^−18^, respectively, Fisher’s exact test, Fig. 5e, Supplementary Data 8). A comparative analysis with the mouse embryonic transcriptional profiles showed significant gene signatures shared between NOR nC7 and nC9 clusters with both, mouse embryonic chromaffin and sympathoblast populations (FDR < 0.01, Fisher’s exact test, Fig. 5b, c, Supplementary Fig. 6a, Supplementary Data 8). In support of these findings, neuroblastoma NOR clusters presented a high signature score (measuring transcriptional resemblance, see “Methods”) for bridge, sympathoblast and chromaffin cells in mouse and human developing adrenal glands, and for human postnatal chromaffin cells (Fig. 6a–c). Furthermore, the significant specific gene signatures of NOR nC7 and nC8 clusters were significantly associated with a better outcome in a large neuroblastoma cohort (i.e., Bonferroni-corrected *p*-value < 0.01, 498 cases in SEQC^17^, see “Methods”, Fig. 6d).

In contrast to the neuroblastoma NOR cells, the undifferentiated neuroblastoma clusters (nC3, nC2) did not share specific gene signatures with mouse embryonic and postnatal sympathoblast nor chromaffin populations (Fig. 5b–e). The nC3 cluster, however, shared a marginally significant number of specific-signature genes with cycling populations in the human fetal gland (i.e., cycling sympathoblast and chromaffin, FDR < 0.05, Fisher’s exact test, Fig. 5e). These were nonetheless only cell-cycle related genes (i.e., *ASPM*, *BUB1*, *CENPE*, *CLSPN*, and *ESCO2*, Supplementary Data 8).

Importantly, and in contrast to the fetal adrenal gland comparison, the neuroblastoma nC3 cluster shared a specific gene signature with the human cholinergic progenitor population (hC1) identified in postnatal human adrenal gland (FDR < 0.01, Fisher’s exact test, Fig. 5d, Supplementary Data 8). In addition, nC3 and hC1 shared a significantly high expression (FDR<0.01, Welch’s *t*-test) of *NTRK2*, mesenchymal (i.e., *COL1A2*, *COL6A3*, *COL12A1*), migratory (i.e., *CLDN11*, *DOCK7*), and progenitor genes (i.e., *BCL11A*, *ERBB3*, *RTTN*, *TP63, ASXL3, POU6F2*, and *SOX6*, Supplementary Data 2, Supplementary Data 3 and 6). Furthermore, the undifferentiated neuroblastoma cluster (nC3) constituted a larger proportion of cells in high-risk samples in both the 11 nuc-Seq-samples and in a deconvolved cohort of 172 patients (FDR < 0.01, Chi-square test, Supplementary Fig. 4), and its specific-signature genes have a marginally higher expression average in patients with a lower survival probability in a larger cohort (i.e., Bonferroni-corrected *p*-value < 0.05, 498 cases in SEQC^17^, see “Methods”, Fig. 6d).

Recently, embryonic multipotent Schwann cell precursors (SCP) have been suggested to be a potential source of neuroblastoma origin^6^. However, embryonic SCPs in both, human fetal and mouse, shared only specific gene signatures with neuroblastoma stroma clusters MSC nC1 and endothelial nC4 (Fig. 5c, d, Supplementary Fig. 6a, Supplementary Data 8). A detailed look into their shared gene signatures suggests a high expression of genes related to cell adhesion and motility (i.e., GO term, FDR < 0.05, Fisher’s exact test) and did not include SCP lineage markers (i.e., *SOX10, S100B, FABP7*, or *PLP1*, Supplementary Data 8).

We further analyzed in detail how the transcriptional profiles of the different neuroblastoma clusters were associated with the survival and age at diagnosis in a larger cohort (i.e., 498 cases in SEQC^17^, Fig. 6e–h, Supplementary Data 9). We compared the specific gene signatures from each neuroblastoma cluster with genes in 498 patient samples with (1) differential expression by risk groups (Fig. 6e), (2) significant correlation with age-at-diagnosis (Fig. 6f), and (3) differential expression by survival outcome (Fig. 6g as detail in “Methods”). Genes in the specific signatures from endothelial nC4, macrophages nC6, undifferentiated nC3, and NOR clusters (nC7, nC8, and nC9) were significantly enriched in both low/intermediate risks and better survival cases (FDR < 0.01, Fisher’s exact test, Fig. 6e–g, Supplementary Data 9). Nevertheless, genes in the specific signatures from undifferentiated nC3 and NOR nC9 clusters were also significantly enriched in both high-risk and worst survival patients (FDR < 0.01, Fisher’s exact test, Fig. 6e–g, Supplementary Data 9). Remarkably, a more significant directly correlation was observed for the specific gene signature of the undifferentiated nC3 cluster with age at diagnosis (i.e., they are more likely to be expressed in later ages, FDR = 8.12 × 10^−31^, Fisher’s exact test), while a more significant inversely correlation was found for the NOR nC9 cluster (FDR  < 1 × 10^−50^, Fisher’s exact test, Fig. 6f, Supplementary Data 9).

A detailed analysis of the specific gene signatures for the undifferentiated nC3 and NOR nC9 clusters revealed that the two cell populations feature different gene programs that can be either (1) directly correlated with age at diagnosis and associated with poor survival (more significant in nC3 than in nC9), or otherwise (2) inversely correlated with age at diagnosis and resulting in better survival (more significant in nC9 than in nC3, FDR < 0.01, Fisher’s exact test, Fig. 6h, Supplementary Data 9). The genes inversely correlated with age at diagnosis, and also those resulting in better survival, are significantly associated with cell adhesion and differentiation for both nC3 and nC9 clusters (GO term, FDR < 0.01, Fisher’s exact test, Supplementary Fig. 6d–g, Supplementary Data 10). Oppositely, genes in NOR nC9 associated with a later age at diagnosis and worst survival are significantly enriched in translation, RNA processing, and splicing, while genes in the undifferentiated nC3 cluster associated with worst survival are enriched in DNA damage and repair and cell cycle, and those associate with a later age of diagnosis are enriched in cell motility (GO term FDR < 0.01, Fisher’s exact test, Supplementary Fig. 6d–g, Supplementary Data 10).

We further investigated the commonalities of human postnatal adrenal gland and neuroblastoma clusters, and performed a comparative analysis based on the detection of matching mutual nearest neighbors^22,23^ (Fig. 7a, b). We used human adrenal gland for the projection of cell states onto neuroblastoma data (i.e., query). In agreement with the identified significant shared gene signatures (shown in Fig. 5d), human postnatal chromaffin (hC4) population matched to the malignant NOR clusters (nC5, nC7, nC8, and nC9), whereas the adrenal gland progenitor population (hC1) matched the malignant undifferentiated neuroblastoma clusters nC2 and nC3 (Fig. 7a, b). Populations of endothelial, macrophages and mesenchymal cells (i.e., stroma cells) in neuroblastoma have a large number of cells with a high state probability of (i.e., best transcriptional match using joined labeling) to similar populations in human adrenal gland.

In addition, we interrogated the relationships between embryonic mouse adrenal anlagen, neuroblastoma, and healthy human postnatal adrenal cell populations. We performed an Euclidean distance-based analysis of the transcriptional similarities between all populations, excluding cortex and immune cells (Fig. 7c, d). This analysis suggested that these populations could be clustered into four groups, namely (1) (nor-) adrenergic, (2) undifferentiated, (3) mesenchymal, and (4) endothelial cells (Fig. 7c). Consistently with the identified significant shared gene signatures (shown in Fig. 5b–d), a statistical analysis of these Euclidean distances (Fig. 7d) indicated that (1) SCPs, glial, endothelial, and mesenchymal cells from human and mouse adult and embryonic E12–E13 populations share a similar transcriptional profile, (2) human adrenal progenitor cluster hC1 resemble that of the neuroblastoma undifferentiated populations nC2 and nC3, and (3) chromaffin cells from human and mouse (postnatal and embryonic) have a similar transcriptional profile, that resemble the neuroblastoma noradrenergic clusters nC5, nC7, nC8, and nC9.

## Discussion

With the aim of understanding the transcriptional basis of the clinical heterogeneity in neuroblastoma, we analyzed 3212 high-quality single cell/nuclei from 11 tumors from patients across different risk groups. We sequenced to an average of ~710,000 reads per nuclei/cell, and compared them to 3456 human and mouse developing and postnatal AG cells sequenced to similar depths. While a larger number of cells could provide a more comprehensive view of the different cell types within the tumors, the large sequencing depth combined with full-length coverage offers higher sensitivity. A recent study compared the results obtained with Smart-Seq2 and 10X Genomics (10X) and concluded that the detection sensitivity was higher in Smart-seq2 than 10X. In particular, the gene capture rate of 20 cells sequenced with Smart-Seq2 is comparable to that of 1000 cells with 10X^24^. Taking advantage of the enhanced sensitivity offered by Smart-Seq2, we consistently recovered the same populations of cells in neuroblastoma tumors of the same risk groups, and compared them with postnatal and developing mouse and human adrenal glands.

The way chromaffin cells are repopulated in the postnatal adrenal gland remains unknown. While during mouse embryonic development, multipotent Schwann cell precursors (i.e., SCPs)^6,11^ and sympathoadrenal progenitors^7^ can give rise to both chromaffin cells and sympathoblasts, their postnatal existence has not been demonstrated. In this study, we identified a population of cells unique to postnatal human adrenal gland that presented precursor features of chromaffin cells, namely (1) the expression of progenitor and migratory markers (i.e., *NTRK2*, *ERBB3*, *BCL11A*, *ASXL3, RTTN*, *SOX6*, *DOCK7*, *LAMA3*, *SEMA3E*, and *CLDN11*), (2) a high differentiation potential, and (3) a computed (by RNA velocity) directionality of the in vivo transitions from progenitor to chromaffin cells. Both human progenitor and chromaffin cells express the cholinergic receptor nAChRs α7 (encoded by the nicotinic acetylcholine receptor gene *CHRNA7*), suggesting that progenitor cells are of cholinergic nature. nAChRα7 is a critical component for the cholinergic system mediating ligand-gated ion channel activation of calcium-dependent signaling pathways^25^, and is involved in the response to cortisol and oxidative stress^26,27^. As this postnatal progenitor population of chromaffin cells does not express the SCP markers *SOX10*, *S100B*, nor *FOXD3* and is not present in the mouse adrenal gland, it is possible that it is unique to humans. RNAscope ISH for markers characteristic of this progenitor population at different ages in human postnatal adrenal glands validated their existence and suggested that this population declines with age.

Comparative analysis of the transcriptional profiles of postnatal human adrenal glands and neuroblastoma tumors of different clinical risk groups revealed that the identified postnatal cholinergic progenitor population (hC1) had a remarkable transcriptional resemblance to the undifferentiated cluster (nC3) characteristic of high-risk neuroblastoma. Both populations, high-risk undifferentiated cluster (nC3) and progenitor population in postnatal gland (hC1) shared a significantly high expression of neurotrophic receptor *NTRK2* (encoding TRKB*)*, mesenchymal (i.e., *COL1A2*, *COL6A3*, and *COL12A1*), migratory (i.e., *LAMA3*, *CLDN11*, and *DOCK7*), and progenitor genes (i.e., *BCL11A*, *ERBB3*, *RTTN*, *TP63, ASXL3, POU6F2*, and *SOX6)*. In contrast, the transcriptional profile of the clusters identified in low-risk neuroblastoma did not resemble the human progenitor population identified in postnatal gland, instead they showed a noradrenergic (NOR) transcriptional signature (i.e., *NTRK1, CHGA, CHGB, DBH*, *TH*, and *PHOX2B*) matching the transcriptome of human and mouse postnatal chromaffin cells, as well as fetal human (8–14 PCW) and mouse embryonic (E13) sympathoblast and chromaffin populations.

Furthermore, our results indicate that the high-risk-associated cell cluster (nC3) has a transcriptional program that changes with age-at-diagnosis and is correlated with lower survival probabilities of patients. This program favors the expression of genes associated with cell motility and metastasis in older patients and with replication stress (i.e., DNA repair and cell cycle) in worst outcome cases. Oppositely, the low-risk-enriched NOR cell clusters presented transcriptional programs associated with greater survival probabilities and younger ages at diagnosis. Within these clusters, the NOR nC9 was an exception as it also showed a transcriptional program that presented a worst outcome in correlation with age. Remarkably, this program is associated with RNA-(mis-)splicing and not with replication stress. Our results suggest that at a younger age (at time of diagnosis) the expression of cell adhesion and differentiation genes from NOR- (and to less extent undifferentiated-) cells signals a better survival probability. Oppositely, a higher expression in older patients of RNA splicing genes from NOR cells, and to a larger extent of replication-stress genes in undifferentiated cells is likely to signal a worst outcome.

In this regard, low-risk neuroblastoma is common in younger children with <18 months of age at the time of diagnosis^1,5^ and *NTRK1* expression (encoding TRKA) is a strong prognostic histological marker for favorable cases, whereas TRKB (encoded by *NTRK2*) is associated with poor outcome^2^. One possibility is that different progenitor populations source neuroblastomas at different ages, thereby accounting for the remarkable clinical heterogeneity that is coupled with age. Thus, age-at-diagnosis is a strong outcome predictor. Specifically, favorable neuroblastomas might originate from embryonic developmental errors when SCPs differentiate to chromaffin and neuroblast, whereas unfavorable neuroblastomas could arise from errors during postnatal development when TRKB+ cholinergic progenitors repopulate chromaffin cells post-natally.

## Methods

### Mice

C57BL/6 mice were kept in rooms with controlled 12-h light/dark cycles, temperature, and humidity with food and water provided. The mice were housed at a maximum of four males per cage and four females per cage. Animal care procedures were in accordance with the guidelines set by the European Community Council Directives (86/609/EEC). Required animal permissions were obtained from the local ethical committee.

### Sample collection and single-cell/nuclei sequencing

Eleven neuroblastoma samples were collected from patients at different ages (0–79 months at diagnosis) and their INSS stages determined following Shimada’s criteria. In particular, three samples were classified as favorable INSS stages (one stage 1, two stage 2/2B), two as favorable widespread stage (4S), and six in intermediate/high metastatic stages (two stage 3, and four stage 4)^4^. According to the INRG clinical and biological criteria five patients were classified as high-risk, one as intermediate risk and five as low-risk, and treated accordingly to local and international protocols^3^. Tumors were genotyped according to Carén et al.^20^, five high-risk genotypes: *MYCN*-amplified and/or 11q-deleted, and six low-risk genotypes: numerical only or other structural (Supplementary Data 1). In addition, three human and five mouse adrenal glands were obtained. All human adrenal samples were collected in conjunction to disease unrelated to pheochromocytoma, paraganglioma, and neuroblastoma. Further details about these samples are included in Supplementary Data 1. Additional post-mortem human adrenal glands for RNAscope analysis were obtained from the NIH Neurobiobank (University of Maryland, Baltimore, MD).

Human samples were collected following surgical resection at the Karolinska University Hospital and processed following the Nuc-Seq protocol^28^ as illustrated in Supplementary Fig. 1. Briefly, nuclei are obtained from deep-frozen tissue after homogenization and filtration, and further FACs sorted in 384-wells plates where cDNA synthesis was conducted with Smart-seq2^29^. Libraries were prepared using the Tn5 transposase tagmentation (Nextera XT), and their quality was assessed with fragment analyzer. High-quality libraries were sequenced using Illumina HiSeq 2500, and further de-multiplexed with deindexer (https://github.com/ws6/deindexer) using the Nextera index adapters and the 384-well layout.

Mouse samples were collected via surgical resection after perfusion and the tissue from each sample was dissociated and sorted in 384-well plates. Sorted cells were lysed and their RNA obtained using the Smart-seq2 protocol^29^, and further processed as described above.

### Read mapping, quality control, and cell clustering

We planned an experimental design that allowed us to obtain and analyze high-quality data^30^. To select high-quality reads we allowed for a hard-clipping of adapters (using CutAdapt 1.13), and excluded reads with less than 20 bases. Additional diagnostics on the reads quality were conducted with FASTAQC (https://www.bioinformatics.babraham.ac.uk/projects/fastqc), cells with reads failing at three or more quality control (QC) tests (among eleven in total including per base- and sequence quality scores, frequency of each nucleotide, GC content, over-represented motifs, and number of duplicated reads) were excluded for further analysis. High-quality reads were mapped with STAR^31^ using 2-pass alignment to have improved performance of *de novo* splice junction reads.

High-quality reads from human samples were mapped to the hg38 human genome version hg38/GRCh38.p12 (released 2019), annotated by the comprehensive annotation of GENCODE 28^32^ as obtained from the UCSC browser^33^ on 25.02.2019. Reads from mouse were mapped to the mm10 mouse genome version mm10/GRCm38.p6 (released 2019), annotated by the comprehensive annotation of GENCODE 18^32^ obtained from the UCSC browser^33^ on 14.01.2019. In both cases, alternative chromosomes were excluded from the annotations, and in the cases of human reads mapping to the mitochondrial genome were excluded (as mitochondrial reads in nuc-Seq can originate from different cells). Gene expression (i.e., read counts allowing ambiguity) was calculated using HTSeq^34^.

To conduct a QC of the cells, different technical and biological features were calculated with QoRT^35^ and Celloline^36^. After an inspection of the features, cells for human adrenal glands expressing at least 3000 and at most 9000 genes, and from mouse adrenals and neuroblastoma expressing at least 2000 and at most 8000 genes were selected for further analysis.

### Expression analysis

Gene expression of cells/nuclei was filtered, transformed, scaled, and standardized accounting for sequence depth, using PAGODA^37^. In particular, error models for individual cells were fitted with parameters robust for noisy data (i.e., min.count.threshold of 2 and at least 5 non-failed measurements per gene). These models were successfully generated for all high-quality cells from mouse (*n* = 1763) and human (*n* = 1322) adrenal glands, and (*n* = 3212) in neuroblastoma. PAGODA clusters cells based on non-redundant significant aspects of transcriptional heterogeneity. The resulting hierarchical clustering was cut by height initially to maximize the silhouette value^38^, and further adjusted to increase the resolution of the defined clusters (i.e., cell/nuclei populations). The standardized expression for mouse E12 and E13 adrenal anlagen (i.e., expression magnitude) was obtained from the PAGODA rds application from Furlan et al.^6^ kindly provided by the authors.

The standardized gene expression from PAGODA (i.e., expression magnitude) was used to determine the genes (A) significantly upregulated in each cluster, and (B) significantly and uniquely upregulated in each cluster (i.e., specific gene signature). In particular, for a given population, the group of genes significantly upregulated in each cluster (i.e., A) is determined as the genes whose average expression is significantly higher than the average expression of cells in all the other clusters. In contrast, the group of genes significantly and uniquely upregulated in each cluster (i.e., B: specific gene signature) is defined as the group of genes whose average expression is significantly higher than each and all of the other clusters’ averages. This significance was calculated using Benjamini–Hochberg multiple test correction on Welch’s *t*-test generated *p*-values, with a FDR threshold of 0.01.

### RNAscope in situ hybridizations

Gene expression in specific clusters was validated using RNAscope. RNAscope of fresh-frozen tissue was performed according to ACD Bio’s manual using the RNAscope Multiplex Fluorescent Detection kit version 2. Probes targeting human-specific genes *NTRK2*(402621-C1, 402621-C2), *CLDN11*(525671-C3), *TH*(441651-C1, 441651-C2, 441651-C3), *CHRNA7*(310101-C1), *CYP11B2*(592851-C2), *RSPO3*(429851-C3), *SOX10*(484121-C2), *ALK*(311841-C1), *MYCN*(417501-C3), *NTRK1*(402631-C1), *PDGFRA*(604481-C1), *LGR5*(311021-C2, 311021-C3), *PRRX1*(497571-C3), *PHOX2B*(567701-C2), and *DBH*(545791-C1) were used.

### Cell cluster analysis

We calculated the under- and over-representation of cells in clusters for neuroblastoma samples, INSS stages, and risk groups, with two-sided Chi-square tests using Yates adjustment and corrected with the Benjamini–Hochberg approach and a FDR threshold of 0.01. In particular, Chi-square tests evaluated the hypothesis that a given case study (risk group for instance) presented a significantly larger or lower proportion of cells in a given cell cluster.

The enrichment of gene ontology (GO) terms were calculated for the significantly upregulated genes in each cluster using a Benjamini–Hochberg multiple test correction on one-sided Fisher’s exact test *p*-values. The same approach was taken to determine the enrichment of significantly upregulated genes defining (1) mesenchymal (MES) and adrenergic (ADR) cell types by van Groningen et al.^16^, and (2) sympathetic noradrenergic (Group 1) and neural-crest cell-like (Group 2) by Boeva et al.^15^. In particular, Fisher’s exact tests evaluated the hypothesis that genes significantly upregulated in a given cell cluster were significantly enriched in a gene set of interest. To conduct inter-species comparisons, only the 1:1 human:mouse orthologues annotated by ENSEMBL version GRCh38.p12^39^ were considered.

To compare the clusters among the various study cases, four different approaches were taken. In the first approach, a comparison of specific gene signatures was conducted following the gene enrichment approach described above, using a FDR threshold of 0.01 and a minimum number of shared genes of 10 (marginally significant results with FDR<0.05 are included in the supplementary data). In the second approach (as displayed in Fig. 7), the average standardized expression (i.e., expression magnitude calculated with PAGODA) from each population was computed and further quantile-normalized using the limma package^40^. Further, Euclidean pairwise distances were calculated for cell populations using their quantile-normalized average gene expression values, and a hierarchical clustering was conducted using Ward’s-2 distance with hclust package and supported with the approximately unbiased *p*-value from the pvclust package^41^. In addition a tSNE was built with the Rtsne package (https://github.com/jkrijthe/Rtsne).

For the third and fourth approaches, the raw gene counts were filtered, normalized, and rescaled using Satija’s pipeline^42^. With these values, we conducted as a third approach an asymmetrical cluster imputation of NB cells using as reference human AG- cells with Seurat version 3^43^. In particular, genes expressed in more than 5 cells, expressed in at least 2 genes were selected. Further processing was conducted using the top 2000 most variable genes after variance transformation and dimension reduction. Finally, as a fourth approach, we calculated a signature score for reference genes significantly upregulated in clusters of interest using Scanpy^44^. This signature score calculates the average expression of the reference genes per 10,000 per cell + 1, minus the average of a (*n*) randomly selected set of genes per 10,000 per cell + 1 (where *n* = max [# reference genes, 50]). The signature score provides an estimation of the transcriptional resemblance of each cell to each reference cluster of interest.

The similarity between clusters of (1) postnatal and fetal^14^ human adrenal glands, and (2) neuroblastoma in this study and from the GOSH cohort^13^, were estimated by two different approaches: (1) computing the enrichment of genes in the specific signatures for genes in the reference clusters (i.e., fetal adrenal gland and GOSH cohort), and (2) computing the signature score for the same references. Gene enrichments were computed with Benjamini–Hochberg corrected one-sided Fisher’s exact tests. Reference gene sets for human fetal adrenal gland were obtained from the differentially expressed genes (with an adjusted *p* < 0.01) in five sympathoadrenal cell types in fetal adrenal glands (i.e., SCP, cycling, and non-cycling sympathoblast/chomaffin cells) reported in Dong et al.^14^. The original cluster annotations are debated and the included labels in this study correspond to those given by Kildisiute et al.^45^, and Bedoya-Reina and Schlisio^46^. Reference gene sets for the GOSH cohort were obtained from the algorithmc markers of different cell population in eight neuroblastoma tumors sequenced by 10X and reported in Kildisiute et al.^13^.

### Analysis of bulk RNA-seq neuroblastoma samples

For gene sets of interest (i.e., target genes), Kaplan–Meier curves were calculated using the 498 SEQC database^17^ and the tools available in the R2 database (http://r2.amc.nl). Specifically, the average standardized expression (*z*-score) for target genes in the 498 SEQC database was calculated, and further classified into two patient groups using the “scan modus” in the KaplanScanner tool. The event free-survival probability was estimated with a log-rank test between these two groups and corrected with the Bonferroni approach. Only specific gene signatures with more than two genes were studied (i.e., specific gene signature from NOR nC5 cluster was excluded as *n* = 2).

Sets of genes significantly (1) upregulated in patients classified in high- (*n* *=* 6696) or non-high- (i.e., low/intermediate, *n* = 8158) risk groups, and (2) directly (*n* = 4572) or inversely (*n* = 3982) correlated with age at diagnosis, were obtained with the GeneSelector tool in R2 for the 498 SEQC database. RPMs were log2-transformed and the difference between risk groups was calculated for each gene using FDR-corrected ANOVAs. Pearson correlations were computed between gene expression and age at diagnosis with FDR-corrected *p*-values signaling the chances of obtaining the correlation coefficient in an uncorrelated dataset. Similarly, gene sets with a significantly higher expression in poor (i.e., worst, *n* = 9049) or better (*n* = 5842) survival cases were retrieved with the Kaplan–Meier Scaner Pro tool, using FDR-correct *p*-values obtained with log-rank tests between groups of patients with different event free-survival probabilities. Significant genes were selected with a FDR threshold of 0.01. Gene enrichment was further calculated with one-sided Fisher’s exact tests corrected with the Benjamini–Hochberg approach.

To calculate the proportion of cells of each neuroblastoma cluster in a larger cohort, 172 TARGET neuroblastoma samples (National Cancer Institute TARGET, dbGap Study Accession: phs000218.v16.p6.) were deconvolved using the reference-based decomposition model in BisqueRNA with the default settings^47^. In comparison with other popular methods, deconvolution by this BisqueRNA has been shown to produce the closest mean estimations to the cell proportions obtained by single nuclei-RNA^47^. Bulk expression from each sample was decomposed into the 10 neuroblastoma cell types using as reference 42,215 genes present in the annotation of both bulk and single-nuclei, filtering 1676 zero-variance and 383 unexpressed genes. To make an accurate deconvolution, bulk-sequence raw data obtained for the 172 TARGET neuroblastoma samples were pre-processed and quantified in a similar way as the single-nuclei data. Briefly, reads were hard-clipped for adapters and low-quality calls, and reads with <20 bases were excluded. High-quality pair-reads were mapped with STAR^31^ using 2-pass alignments to the hg38 human genome version hg38/GRCh38.p12 (released 2019) annotated with the comprehensive annotation of GENCODE 28^32^, and further quantified with HTSeq^34^. The expected (i.e., proportional) number of cells per cluster for each deconvolved sample was computed as the product of the predicted cluster percentage, and the proportional number of cells (*c*) in the TARGET NB samples (*c* = [(average number of nuclei from 11 nuc-Seq neuroblastomas in the same risk group) × (number of TARGET NB samples in the risk group)]. The significance of the expected (i.e., proportional) number of cells was computed with Benjamini–Hochberg corrected two-sided Chi-squares tests with Yates’ adjustment.

### Genome rearrangement analysis

Genome rearrangements of cells (i.e., copy number variants) were determined using inferCNV v1.4.0 of the Trinity CTAT Project (https://github.com/broadinstitute/inferCNV). InferCNV was computed with the gene expression for seven samples (out of the initial 11: K87, K10, 23, K55, K3, 19, and K6) that presented cells in the immune clusters: macrophages (nC6) and/or T cells (nC10). Arrangements in these cells were used as a non-malignant reference. Copy number variations (CNVs) were calculated from moving averages of 101-genes’ windows with the default hidden Markov model. Tumor cells were simulated to have CNVs with probabilities (*p*) that matched six possible states, from a chromosomal segment complete loss to a gain of more than 2 copies (1 = complete loss, 2 = loss of one copy, 3 = neutral, 4 = addition of one copy, 5 = addition of 2 copies, 6 = addition of more than 2 copies). These probabilities were used to estimate the likelihood of observing a rearrangement (total sum of *p*) and to select the most likely state (maximum *p*). CNVs with a (Bayesian) likelihood value of 0.1 (probability of being true rearrangements >0.9) were selected for further analysis.

For each sample independently, rearrangements (i.e., expected states) in each region of interest (i.e., 1p loss, 11q loss, and 17q gain) were computed for each cell as the average of the most likely state (as either gain or loss) for the overlapping CNVs. The significance for gains or losses in each cluster was calculated by testing the hypothesis that the number of cells with the rearrangement was significantly higher than expected, using a Benjamini–Hochberg corrected Fisher’s exact test.

Rearrangements for tumor samples were confirmed using Affymetrix 250K arrays^20^. The samples were compared in silico to constitutional DNA from healthy individuals. The array experiments were performed following the protocol provided by the supplier (Affymetrix, Inc., Santa Clara, CA; from 2016 sold by Thermo Fisher Scientific, Waltham, MA). Briefly, total genomic DNA (250 ng) was digested with NspI and ligated to adapters. After ligation, the template was subjected to PCR amplification using a generic primer that recognizes the adapter sequence. The amplified DNA was fragmented with DNase I, labeled with biotin, and hybridized to a GeneChip Human Mapping 250 K NspI array. The hybridized probes were washed using the Affymetrix Fluidics Station 450 and marked with streptavidin-phycoerythrin. The arrays were scanned using a confocal laser scanner, GeneChip Scanner 3000 (Thermo Fisher Scientific, Waltham, MA). Primary data analysis was performed processed using GDAS software (Thermo Fisher Scientific, Waltham, MA) and CNVs were estimated using CNAG (Copy Number Analyzer for Affymetrix GeneChip Mapping arrays) software, version 3.0 (GenomeLaboratory, Tokyo University, http://www.genome.umin.jp).

### Cell velocities and differentiation potential analysis

To calculate the differentiation trajectory of cells, the percentage of splicing and unspliced genes per cell was estimated with velocyto^18^. Further, the cell velocities were calculated using scVelo^18,19^. To obtain high confidence velocities (as confirmed by visual inspection of confidence scores) for human adrenal gland we selected genes with a minimum number of shared read counts of 40, and built the velocities using the top 600 genes. For mouse adrenal gland, these parameters were 3000 and 1000, respectively. Further, the gene transition over the pseudotime (representing the cell’s internal clock, and the approximate time of cell differentiation) was determined using a multiple kinetic regimes, and illustrated for the selected populations of interest. The differentiation potential of cells was determined with Palantir^48^ using the default parameters, and as starting cell the one with the highest expression of the precursor marker *ERBB3*. This approach uses entropy to estimate cell plasticity in modeled trajectories of cells fates, so that cell plasticity increases with entropy.

### Ethical considerations

All animal experiments complied with the ethical regulations for animal testing and research and were performed according to Swedish guidelines and regulations, and the ethical permit 7694-2017 was granted by “Stockholms Norra djurförsöksetiska nämnd, Sweden” (Jordbruksverket). Human samples were collected under the ethical permits from Stockholm Regional Ethical Review Board and the Karolinska University Hospital Research Ethics Committee in agreement with the Declaration of Helsinki. Ethical permits for neuroblastoma samples (009/1369-31/1 and 03-736) were issued to Professor P. Kogner and permits for adrenal gland samples (KI 2007/069 and KI 2001/136) were issued to Professor C. Larsson. All adult human adrenal samples were removed at surgery from three adult patients following written informed consent (*n* = 3). All 11 neuroblastoma tumor samples from Swedish patients were collected after obtaining verbal or written consent from parents or guardians depending on IRB requirements at the time of sampling. All samples were collected and analyzed according to permits approved by the Karolinska Institutet and the Karolinska University Hospital ethics committees (reference numbers 03-736 and 2009/1369-31/1) in agreement with the Declaration of Helsinki. Sampling was performed during clinical routine procedures, treatment and management were performed according to established national and international protocols, and hospital records were used to retrieve clinical data. Post-mortem human adrenal glands for staining’s were obtained from the NIH Neurobiobank (University of Maryland, Baltimore, MD) under the same ethical permit from Stockholm Regional Ethical Review Board and the Karolinska University Hospital Research Ethics Committee (KI 2007/069 and KI 2001/136).

### Reporting summary

Further information on research design is available in the Nature Research Reporting Summary linked to this article.

## Supplementary information

Supplementary Information

Description of Additional Supplementary Files

Supplementary Data 1

Supplementary Data 2

Supplementary Data 3

Supplementary Data 4

Supplementary Data 5

Supplementary Data 6

Supplementary Data 7

Supplementary Data 8

Supplementary Data 9

Supplementary Data 10

Reporting Summary

## Data Availability

Interactive analysis of the results can be conducted in https://oxygen.mtc.ki.se/nc_nb_2021.html. Raw sequences for human adrenal gland are available in the Synapse ID project syn22301662. Raw sequences for human adrenal gland sample 6657 are available with the Synapse IDs syn22302285 and syn25189163. Raw sequences for human adrenal gland sample 6435 are available with the Synapse IDs syn22301836. Raw sequences for human adrenal gland sample 16-D are available with the Synapse IDs syn22301667. Raw sequences for neuroblastoma are available in the Synapse ID project syn22302605. Raw sequences for neuroblastoma sample K87 are available in the Synapse ID syn22307346. Raw sequences for neuroblastoma sample K6 are available in the Synapse ID syn22306928. Raw sequences for neuroblastoma sample K55 are available in the Synapse ID syn22306349. Raw sequences for neuroblastoma sample K47 are available in the Synapse ID syn22305928. Raw sequences for neuroblastoma sample K40 are available in the Synapse ID syn22305408. Raw sequences for neuroblastoma sample K3 are available in the Synapse ID syn22304935. Raw sequences for neuroblastoma sample K2 are available in the Synapse ID syn22304482. Raw sequences for neuroblastoma sample K14 are available in the Synapse ID syn22304038. Raw sequences for neuroblastoma sample K10 are available in the Synapse ID syn22303649. Raw sequences for neuroblastoma sample 23 are available in the Synapse ID syn22303256. Raw sequences for neuroblastoma sample 19 are available in the Synapse ID syn22302820. Raw sequences for mouse adrenal gland are available in the Synapse ID project syn22308005. Raw sequences for mouse adrenal gland sample 6801 are available in the Synapse ID syn22308008. Raw sequences for mouse adrenal gland sample 6802 are available in the Synapse ID syn22308616. Raw sequences for mouse adrenal gland sample 3431 are available in the Synapse ID syn22309301. Raw sequences for mouse adrenal gland sample 3432 are available in the Synapse ID syn22310231. Raw sequences for mouse adrenal gland sample I1 are available in the Synapse ID syn22309843. The National Cancer Institute TARGET publicly available data used in this study are available in the dbGap database under accession code phs000218.v16.p6. The SEQC publicly available data used in this study are available in the GEO database under accession code GSE62564. The remaining data are available within the Article or the Supplementary Information files.
