# Supplementary material for: Single-nuclei transcriptomes from human adrenal gland reveal distinct cellular identities of low and high-risk neuroblastoma tumors
Source: Nat Commun. 2021 Sep 7;12:5309. doi: 10.1038/s41467-021-24870-7 (PMC8423786; doi:10.1038/s41467-021-24870-7)
Supplement: Supplementary file 1 — Supplementary Information [file 41467_2021_24870_MOESM1_ESM.pdf]

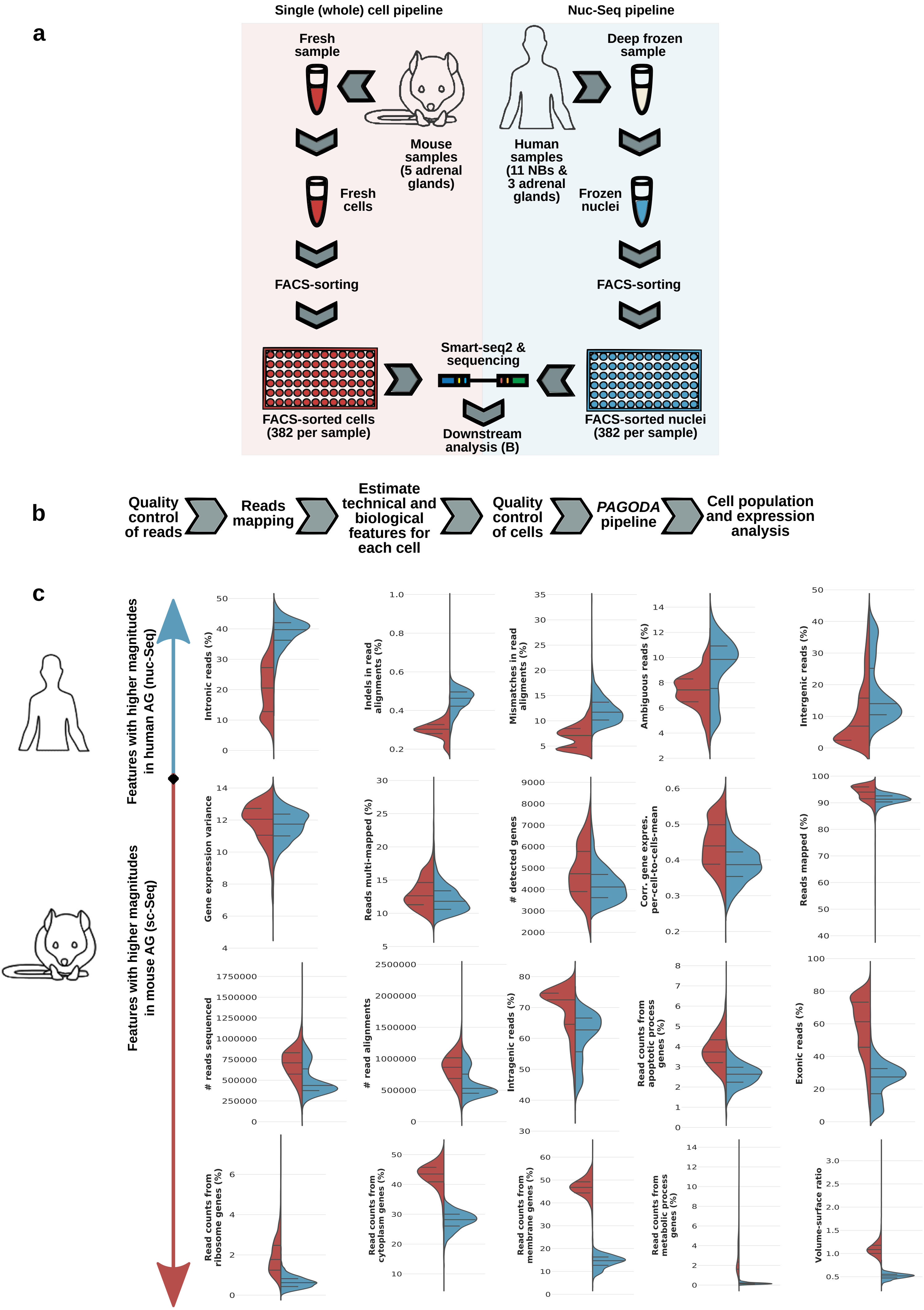
a
Single (whole) cell pipeline
Nuc-Seq pipeline
Fresh sample
Mouse samples (5 adrenal glands)
Fresh cells
FACS-sorting
FACS-sorted cells (382 per sample)
Human samples (11 NBs & 3 adrenal glands)
Deep frozen sample
Frozen nuclei
FACS-sorting
FACS-sorted nuclei (382 per sample)
Smart-seq2 & sequencing
Downstream analysis (B)
b
Quality control of reads
Reads mapping
Estimate technical and biological features for each cell
Quality control of cells
PAGODA pipeline
Cell population and expression analysis
c
Features with higher magnitudes in human AG (nuc-Seq)
Features with higher magnitudes in mouse AG (sc-Seq)
Intronic reads (%)
Indels in read alignments (%)
Mismatches in read aligments (%)
Ambiguous reads (%)
Intergenic reads (%)
Gene expression variance
Reads multi-mapped (%)
detected genes
Corr. gene expres. per-cell-to-cells-mean
Reads mapped (%)
reads sequenced
read alignments
Intragenic reads (%)
Read counts from apoptotic process genes (%)
Exonic reads (%)
Read counts from ribosome genes (%)
Read counts from cytoplasm genes (%)
Read counts from membrane genes (%)
Read counts from metabolic process genes (%)
Volume-surface ratio

Supplementary Figure 1. a-b, Overview of the pipeline used to sample, sequence and analyze single nuclei/cell from mouse and human adrenal glands (AG) and neuroblastoma (NB). a, Samples were collected and processed following sc-Seq [29] and the nuc-Seq protocols [28]. Nuclei (for fresh frozen human samples) and whole cells (for mouse samples) were obtained and FACs-sorted in 384-wells plates. Libraries were prepared with Smart-Seq2 and sequenced with Illumina HiSeq 2500. **b,** High-quality reads and cells were selected for further analysis with PAGODA. After clustering, gene expression for each cluster was determined and further compared with other case studies and reference databases using four different approaches detailed in Methods. Different technical and biological features characterize single(-whole cell, i.e. sc-Seq), and single(-nucleus, i.e. nuc-Seq) sequencing. **c,** Features associated to partial transcript splicing and transcription noise are higher in nuc-Seq than in sc-Seq, including the percentage of intronic, indels in read alignments, and intergenic reads. Oppositely, features associated to (cytoplasmic) mature transcripts are higher in sc-Seq than in nuc-Seq, including percentage of intragenic and exonic reads.

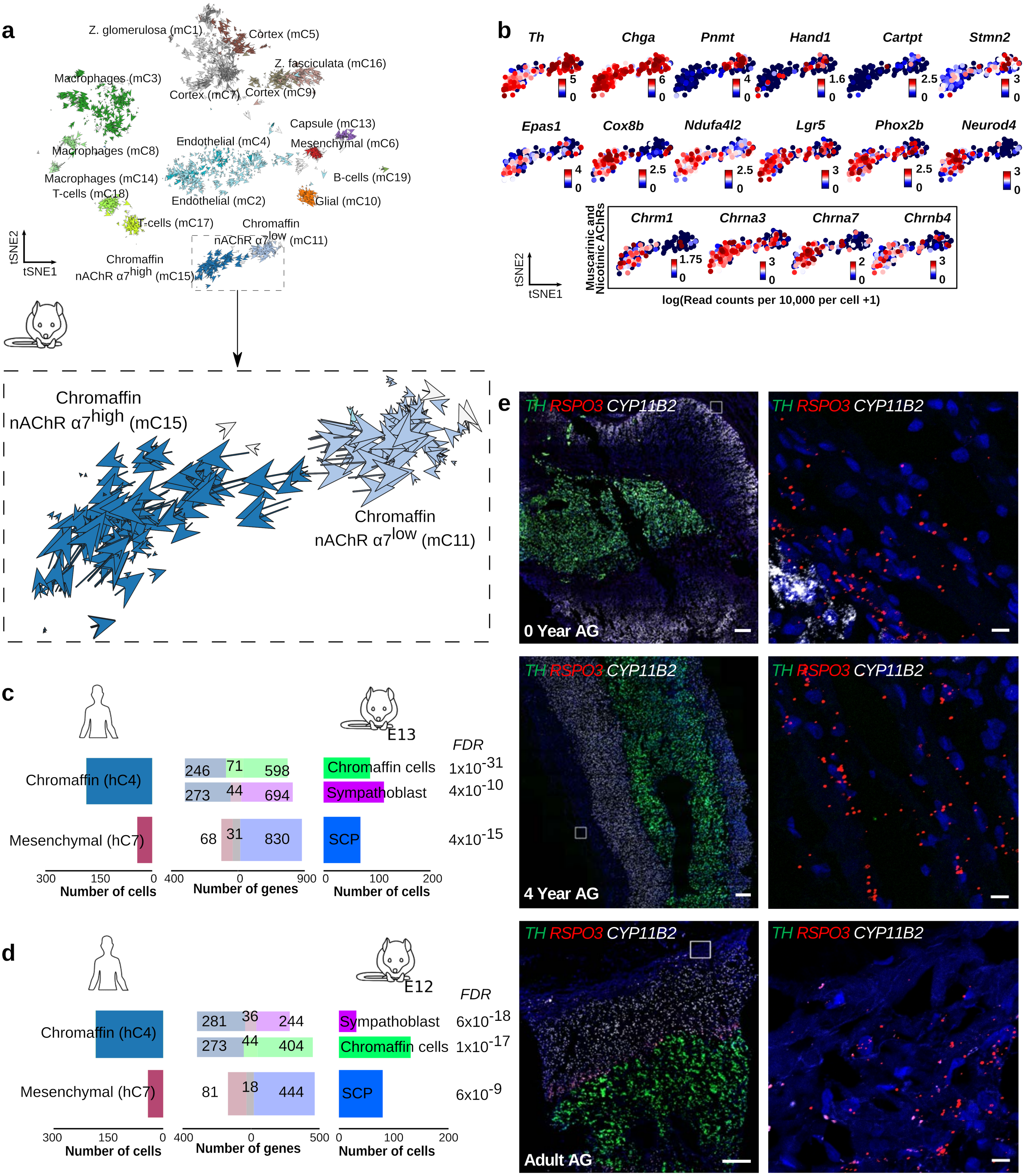
a
Z. glomerulosa (mC1)
Cortex (mC5)
Z. fasciculata (mC16)
Macrophages (mC3)
Cortex (mC7)
Cortex (mC9)
Capsule (mC13)
Endothelial (mC4)
Mesenchymal (mC6)
Macrophages (mC8)
Macrophages (mC14)
T-cells (mC18)
B-cells (mC19)
Endothelial (mC2)
Glial (mC10)
T-cells (mC17)
Chromaffin nAChR α7low (mC11)
Chromaffin nAChR α7high (mC15)
tSNE2
tSNE1
b
Th
Chga
Pnmt
Hand1
Cartpt
Stmn2
Epas1
Cox8b
Ndufa4l2
Lgr5
Phox2b
Neurod4
Muscarinic and Nicotinic AChRs
Chrm1
Chrna3
Chrna7
Chrnb4
log(Read counts per 10,000 per cell +1)
c
E13
FDR
Chromaffin (hC4)
246 71 598
273 44 694
Chromaffin cells 1x10-31
Sympathoblast 4x10-10
Mesenchymal (hC7)
68 31 830
SCP 4x10-15
300 150 0
Number of cells
400 0 900
Number of genes
0 100 200
Number of cells
d
E12
FDR
Chromaffin (hC4)
281 36 244
273 44 404
Sympathoblast 6x10-18
Chromaffin cells 1x10-17
Mesenchymal (hC7)
81 18 444
SCP 6x10-9
300 150 0
Number of cells
400 0 500
Number of genes
0 100 200
Number of cells
e
TH RSPO3 CYP11B2
0 Year AG
4 Year AG
Adult AG

Supplementary Figure 2. Anatomy of human and mouse adrenal gland (AG) revealed from single nuclei/cell analysis. a-b, Chromaffin cells in mouse are grouped in two different clusters characterized by the low and high expression of nAChR α7 (encoded by *CHRNA7*). **a,** Velocity analysis suggests that each group has a different fate and do not inter-convert between them. **b**, A panel of noradrenergic markers is shared between the two population, and others characterize each cluster, particularly the expression in *PNMT* is higher in mC11 in comparison to mC15 (FDR<0.01, Welch's *t*-test). Other genes that have a higher (non-significant) average expression include *CARTPT, ISL1, and HAND1.* Oppositely, the expression of *EPAS1, 1233 COX8B, NDUFA4L2, PHOX2B* and *NEUROD4* is significantly higher (FDR<0.01) in mC15 than in mC11. The expression of a repertoire of Muscarinic and Nicotinic cholinergic receptors is higher in mC15 than mC11 (FDR<0.01, Welch's *t*-test). The bars next to the tSNEs illustrate the expression measured as the logarithm of the read counts per 10,000. **c-d**, Specific signatures significantly shared between human adrenal gland and mouse adrenal anlagen at (**c**) E13 and (**d**) E12 (FDR<0.01, Welch's *t*-test, marginally significant results are included in Supplementary Table 3). **e,** Overview of tile-scanned images (20x) of post-natal human adrenal glands (AG) at indicated age. Scalebar of overview: 200µm, zoom of boxed image (indicating capsule): 10µm. RNAscope *in situ* hybridization for *TH* (green) labeling adrenal medulla, *RSPO3* (red) labeling adrenal capsule and *CYP11B2* (white) labeling adrenal cortex. Nuclei were counter-stained with DAPI (blue). For all RNAscope experiments, the signal distribution patterns and cell morphological features were shown by the different combination of probes and independently reproduced three times on different samples.

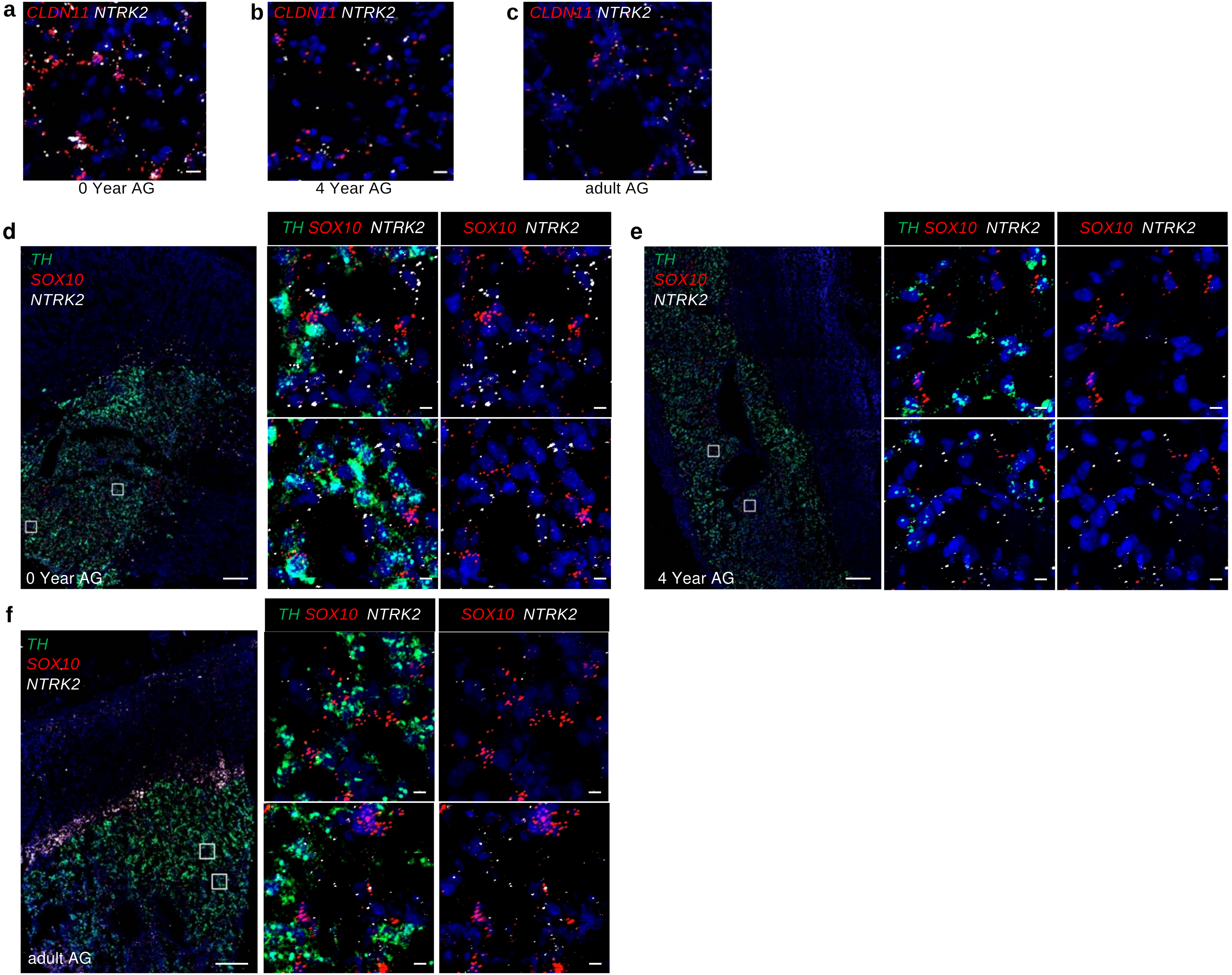
a
CLDN11 NTRK2
0 Year AG
b
CLDN11 NTRK2
4 Year AG
c
CLDN11 NTRK2
adult AG
d
TH
SOX10
NTRK2
0 Year AG
TH SOX10 NTRK2
SOX10 NTRK2
e
TH
SOX10
NTRK2
4 Year AG
TH SOX10 NTRK2
SOX10 NTRK2
f
TH
SOX10
NTRK2
adult AG
TH SOX10 NTRK2
SOX10 NTRK2

Supplementary Figure 3. Location of human cholinergic progenitor (*NTRK2+ CLDN11+)* within the post-natal human adrenal gland (AG). **a-c**, RNAscope ISH zoom images of AG of indicated age as shown in Figure 2 without *TH* (green) channel. *NTRK2* mRNA is shown in white and *CLDN11* mRNA is shown in red. Scalebar: 10µm. **d-f**, Overview images of tile-scanned (20x) post-natal human AG at indicated age. Scalebar of overview: 200µm, zoom of boxed image: 10µm. Adrenal medulla labeled with RNAscope *in situ* hybridization for *TH* (green), *SOX10* (red) and *NTRK2* (white) mRNA and counter stained with DAPI (blue). *NTRK2*+ positive cells were exclusive from *SOX10+* cells. For all RNAscope experiments, the signal distribution patterns and cell morphological features were shown by the different combination of probes and independently reproduced three times on different samples.

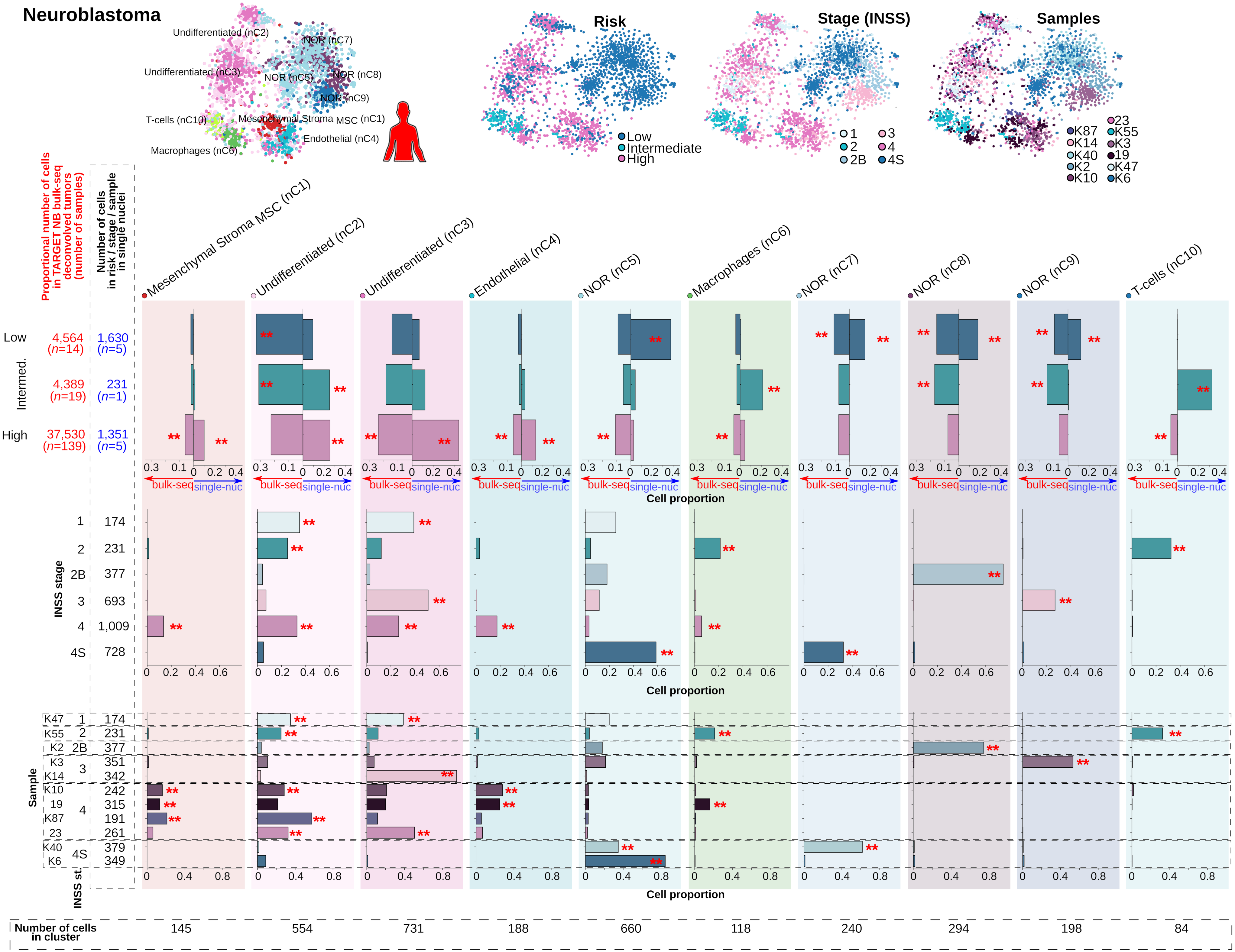

Neuroblastoma
Undifferentiated (nC2)
NOR (nC7)
Undifferentiated (nC3)
NOR (nC5)
NOR (nC8)
NOR (nC9)
T-cells (nC10)
Mesenchymal Stroma MSC (nC1)
Endothelial (nC4)
Macrophages (nC6)
Risk
Low
Intermediate
High
Stage (INSS)
1
2
2B
3
4
4S
Samples
K87
K14
K40
K2
K10
23
K55
K3
19
K47
K6
Proportional number of cells in TARGET NB bulk-seq deconvolved tumors (number of samples)
Number of cells in risk / stage / sample in single nuclei
Mesenchymal Stroma MSC (nC1)
Undifferentiated (nC2)
Undifferentiated (nC3)
Endothelial (nC4)
NOR (nC5)
Macrophages (nC6)
NOR (nC7)
NOR (nC8)
NOR (nC9)
T-cells (nC10)
Risk
Low 4,564 (n=14) 1,630 (n=5)
Intermed. 4,389 (n=19) 231 (n=1)
High 37,530 (n=139) 1,351 (n=5)
bulk-seq
single-nuc
Cell proportion
INSS stage
1 174
2 231
2B 377
3 693
4 1,009
4S 728
Cell proportion
Sample
K47 1 174
K55 2 231
K2 2B 377
K3 3 351
K14 342
K10 4 242
19 315
K87 191
23 261
K40 4S 379
K6 349
INSS st.
Cell proportion
Number of cells in cluster
145
554
731
188
660
118
240
294
198
84

Supplementary Figure 4. Different cell populations are differently represented in neuroblastoma risks groups and INSS stages. Undifferentiated and stroma cell clusters in neuroblastoma (i.e. nC1, nC2, nC3 and nC4) represent a larger proportion of cells in high-risk neuroblastoma, while noradrenergic clusters (i.e. nC5, nC7, nC8, and nC9) represent a larger proportion of cells in low-risk neuroblastoma (top right, single-nuc). 172 TARGET NB bulk-sequenced samples (NB172 NCI TARGET project) in different risks groups were deconvolved to estimate the expected number of cells from each NB cell clusters (top left, bulk-seq). A significant higher number of cells (Benjamini-Hochberg corrected Chi-square tests) was recapitulated in high-risks samples for clusters MSC nC1, Undifferentiated nC3, and Endothelial nC4; and in low-risk for NOR clusters nC7, nC8, and nC9. Cell numbers in deconvolution correspond to the product of the predicted proportion of cells for each cluster, and the proportional number of cells in the TARGET NB samples (calculated as detailed in Methods). ** signals clusters with a significantly larger number of cells than expected (FDR<0.01, two-sided Chi-square using Yates adjustment and corrected with Benjamini-Hochberg). Colors of bars displayed in the risk, INSS stage, and Samples inserts, represent their corresponding neuroblastoma groups by color in the top tSNE inserts.

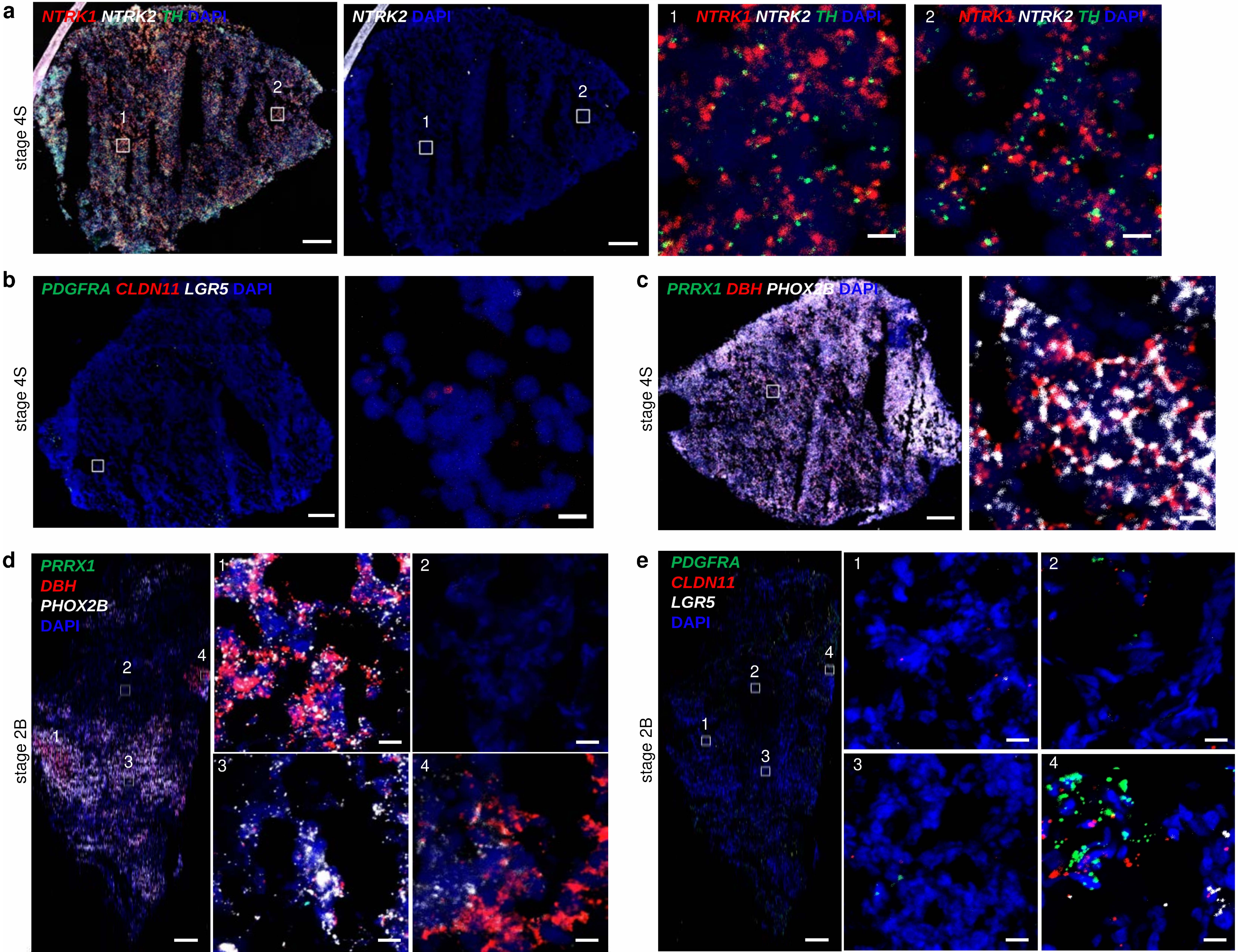

a
NTRK1 NTRK2 TH DAPI
NTRK2 DAPI
1 NTRK1 NTRK2 TH DAPI
2 NTRK1 NTRK2 TH DAPI
stage 4S
b
PDGFRA CLDN11 LGR5 DAPI
stage 4S
c
PRRX1 DBH PHOX2B DAPI
stage 4S
d
PRRX1
DBH
PHOX2B
DAPI
stage 2B
e
PDGFRA
CLDN11
LGR5
DAPI
stage 2B

Supplementary Figure 5. *In situ* transcriptomic images of favorable neuroblastoma 4S and stage 2B. Overview of tile-scanned (20x) favorable neuroblastoma after RNAscope ISH. Scalebar of overview: 200µm; zoom of boxed image: 10µm. **a-c,** RNAscope ISH of low-risk INSS stage 4S neuroblastoma (**a**) for *NTRK1* (red), *NTRK2* (white) and *TH* (green) revealing homogeneous expression of *NTRK1* and *TH* mRNA in the of entire tumor section with no evidence of *NTRK2* positive cells. **b**, RNAscope ISH for *PDGFRA* (green), *CLDN11*(red) and *LGR5* (white) reveals no expression of these mRNAs. **c**, RNAscope ISH for *PRRX1* (green), *DBH* (red) and *PHOX2B* (white) showing homogeneous expression of *DBH* and *PHOX2B* mRNA in the entire tumor with no evidence of *PRRX1* positive cells. **d,** RNAscope ISH in low-risk INSS stage 2B neuroblastoma revealing a more heterogeneous pattern of *DBH* (red) and *PHOX2B* (white) positive tumor region with no evidence of *PRRX1 (green)* labeled cells. **e,** RNAscope ISH for *PDGFRA* (green), *CLDN11* (red) and *LGR5* (white) in adjacent section of (**d**) with no evidence of their expression. Only one small region of the entire tumor (box #4) showed positively labeled cells. For all RNAscope experiments, the signal distribution patterns and cell morphological features were shown by the different combination of probes and independently reproduced three times on different samples in **a-d**, and independently reproduced four times on different samples in **e.**

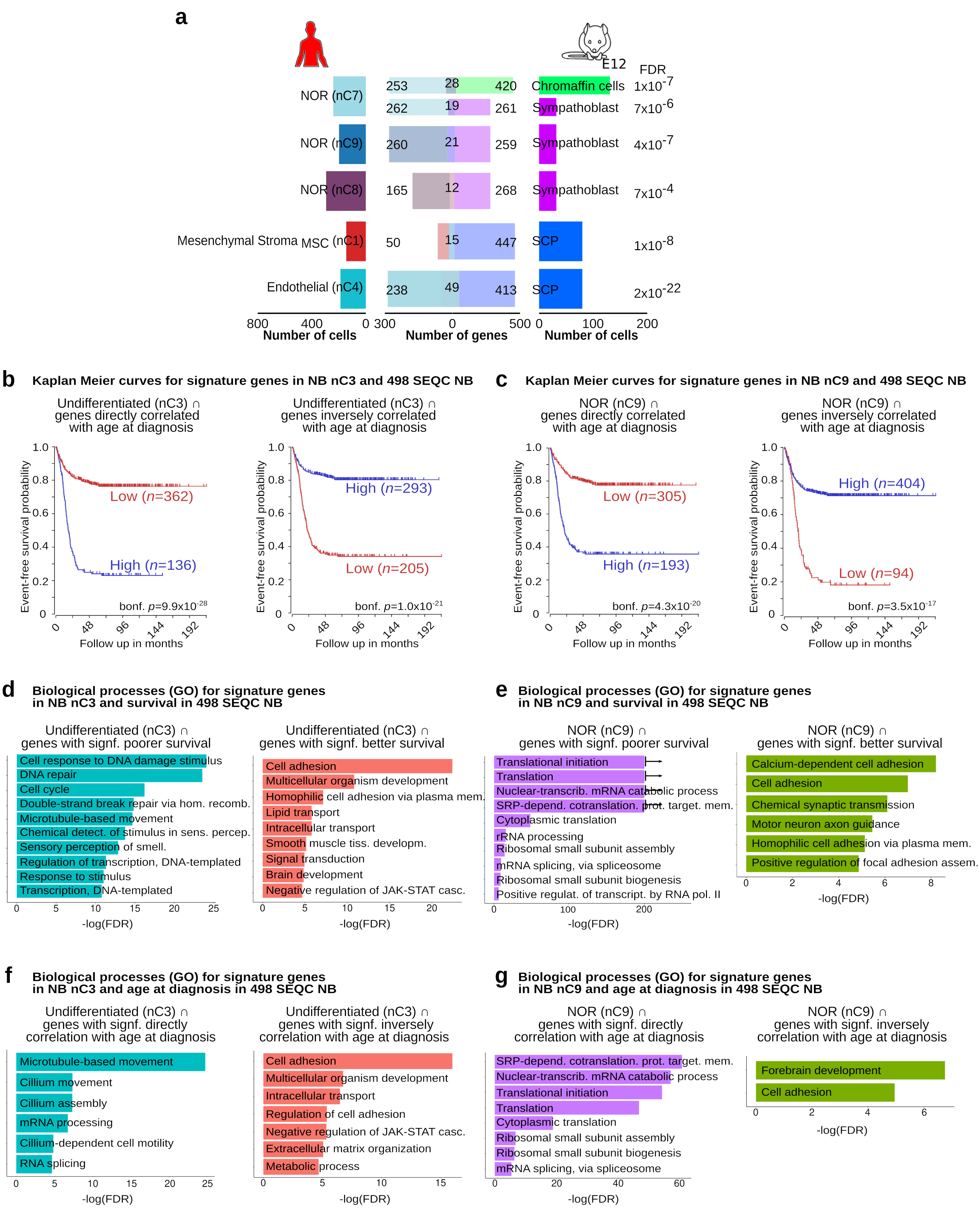

a
E12
FDR
NOR (nC7)
NOR (nC9)
NOR (nC8)
Mesenchymal Stroma MSC (nC1)
Endothelial (nC4)
253 28 420 Chromaffin cells 1x10-7
262 19 261 Sympathoblast 7x10-6
260 21 259 Sympathoblast 4x10-7
165 12 268 Sympathoblast 7x10-4
50 15 447 SCP 1x10-8
238 49 413 SCP 2x10-22
Number of cells
Number of genes
Number of cells
b Kaplan Meier curves for signature genes in NB nC3 and 498 SEQC NB
Undifferentiated (nC3) ∩ genes directly correlated with age at diagnosis
Low (n=362)
High (n=136)
bonf. p=9.9x10-28
Undifferentiated (nC3) ∩ genes inversely correlated with age at diagnosis
High (n=293)
Low (n=205)
bonf. p=1.0x10-21
c Kaplan Meier curves for signature genes in NB nC9 and 498 SEQC NB
NOR (nC9) ∩ genes directly correlated with age at diagnosis
Low (n=305)
High (n=193)
bonf. p=4.3x10-20
NOR (nC9) ∩ genes inversely correlated with age at diagnosis
High (n=404)
Low (n=94)
bonf. p=3.5x10-17
Event-free survival probability
Follow up in months
d Biological processes (GO) for signature genes in NB nC3 and survival in 498 SEQC NB
Undifferentiated (nC3) ∩ genes with signf. poorer survival
Cell response to DNA damage stimulus
DNA repair
Cell cycle
Double-strand break repair via hom. recomb.
Microtubule-based movement
Chemical detect. of stimulus in sens. percep.
Sensory perception of smell.
Regulation of transcription, DNA-templated
Response to stimulus
Transcription, DNA-templated
Undifferentiated (nC3) ∩ genes with signf. better survival
Cell adhesion
Multicellular organism development
Homophilic cell adhesion via plasma mem.
Lipid transport
Intracellular transport
Smooth muscle tiss. developm.
Signal transduction
Brain development
Negative regulation of JAK-STAT casc.
-log(FDR)
e Biological processes (GO) for signature genes in NB nC9 and survival in 498 SEQC NB
NOR (nC9) ∩ genes with signf. poorer survival
Translational initiation
Translation
Nuclear-transcrib. mRNA catabolic process
SRP-depend. cotranslation. prot. target. mem.
Cytoplasmic translation
rRNA processing
Ribosomal small subunit assembly
mRNA splicing, via spliceosome
Ribosomal small subunit biogenesis
Positive regulat. of transcript. by RNA pol. II
NOR (nC9) ∩ genes with signf. better survival
Calcium-dependent cell adhesion
Cell adhesion
Chemical synaptic transmission
Motor neuron axon guidance
Homophilic cell adhesion via plasma mem.
Positive regulation of focal adhesion assem.
-log(FDR)
f Biological processes (GO) for signature genes in NB nC3 and age at diagnosis in 498 SEQC NB
Undifferentiated (nC3) ∩ genes with signf. directly correlation with age at diagnosis
Microtubule-based movement
Cillium movement
Cillium assembly
mRNA processing
Cillium-dependent cell motility
RNA splicing
Undifferentiated (nC3) ∩ genes with signf. inversely correlation with age at diagnosis
Cell adhesion
Multicellular organism development
Intracellular transport
Regulation of cell adhesion
Negative regulation of JAK-STAT casc.
Extracellular matrix organization
Metabolic process
-log(FDR)
g Biological processes (GO) for signature genes in NB nC9 and age at diagnosis in 498 SEQC NB
NOR (nC9) ∩ genes with signf. directly correlation with age at diagnosis
SRP-depend. cotranslation. prot. target. mem.
Nuclear-transcrib. mRNA catabolic process
Translational initiation
Translation
Cytoplasmic translation
Ribosomal small subunit assembly
Ribosomal small subunit biogenesis
mRNA splicing, via spliceosome
NOR (nC9) ∩ genes with signf. inversely correlation with age at diagnosis
Forebrain development
Cell adhesion
-log(FDR)

Supplementary Figure 6. Expression signatures of neuroblastoma cell clusters are associated with patient survival and age at diagnosis. a, Specific gene signatures significantly shared between neuroblastoma and mouse adrenal anlagen at E12. Bar colors represent cell clusters by color in Figure 6. **b-c** Kaplan Meier curves for gene sets with significant differences (Bonferroni corrected [bonf. p], logrank tests) in the survival of 498 SEQC neuroblastoma patients with a low (red) and high (blue) expression [17] for signature genes of the neuroblastoma undifferentiated nC3 and NOR nC9 clusters, directly and inversely correlated with age at diagnosis in patients from the SEQC cohort [17]. A gene enrichment-based approach (Benjamini-Hochberg corrected, Fisher's exact tests) of the specific signature genes for the NOR nC9, and undifferentiated nC3 clusters in 498 SEQC neuroblastoma patients [17], indicates different biological processes (GO) associated with **d-e,** survival and **f-g,** age-at-diagnosis. At most the top ten GO terms with FDR<0.01 are shown. ∩ symbol signifies the intersection between the two gene sets. An arrow next to the bar indicates a FDR less than $1x10^{-50}$.
