# Supplementary material for: Single-nuclei transcriptomes from human adrenal gland reveal distinct cellular identities of low and high-risk neuroblastoma tumors
Source: Nat Commun. 2021 Sep 7;12:5309. doi: 10.1038/s41467-021-24870-7 (PMC8423786; doi:10.1038/s41467-021-24870-7)
Supplement: Supplementary file 2 — Description of Additional Supplementary Files [file 41467_2021_24870_MOESM2_ESM.docx]

**Description of Additional Supplementary Files**

Single-nuclei transcriptomes from human adrenal gland reveal distinct cellular identities of low and high-risk neuroblastoma tumors

Bedoya-Reina O.C.,^1,*,+^, Li W.^1,+^, Arceo M.^1^, Plescher M.^1^, Bullova P^1^, Pui H.^2^, Kaucka M.^3^, Kharchenko P.^4,5^, Martinsson T.^6^, Holmberg J.^7^, Adameyko I.^2,8^, Deng Q.^2^ , Larsson C.^9^, Juhlin C.C.^9^ , Kogner P.^10^, Schlisio S.^1,*^

*Corresponding authors: Oscar C. Bedoya-Reina, Department of Microbiology, Tumor and Cell Biology. Karolinska Institutet. Stockholm, Sweden. E-mail: [oscar.bedoya.reina@ki.se](mailto:oscar.bedoya.reina@ki.se). Susanne Schlisio, Department of Microbiology, Tumor and Cell Biology. Karolinska Institutet. Stockholm, Sweden. E-mail: [susanne.schlisio@ki.se](mailto:susanne.schlisio@ki.se).

+Equally contributing authors

**Supplementary Data Legends**

**Supplementary Data 1.** **List of samples, cells and reads details and statistics for each case study, before and after quality controls.** Extended information of the neuroblastoma samples is included. CGH class: OS=Other segmental, 17q=17q+ without NMA/11q-', NMA=MYCN-amplification. INRGSS stage: L=Localized, L1/L2 (INSS 1,2,3), INRGSS=International Neuroblastoma Risk Group Staging System, INSS=International Neuroblastoma Staging System, CGH=Comparative genomic hybridization.

**Supplementary Data 2. Lists of cured markers from literature used to annotate cell populations in neuroblastoma, and mouse and human adrenal glands (AG), and their specific gene signatures.** Only genes with a significantly high expression in any cluster *x* (i.e. FDR(*sCx>sCo*)≤0.01) are included in the table. For a given case study *s*, FDR(*sCx>sCo*) test the hypothesis that the expression of a gene in cell cluster *x* is higher than in cells from all other clusters (“Each cluster in s: FDR” column); and FDR(*sCx>sCy*) test the hypothesis that the expression of a gene in cell cluster *x* is higher than in cells of cluster *y* (“Each cluster in pairwise comparison *s*: FDR” column). FDRs were calculated with a Benjamini-Hochberg correction on Welch's *t*-tests (as detailed in Methods). Clusters with FDR(*sCx>sCo*)≤0.01 are included in the column “Evidence for high expression in cluster”. All clusters *y* in this column that do not present in pairwise comparisons any FDR(*sCx>sCy*)≤0.01 are included in the column “Evidence for higher expression in cluster”. Specific gene signatures for clusters in human and mouse post-natal adrenal gland, and neuroblastoma were calculated as detailed in Methods.

**Supplementary Data 3. Genes significantly up-regulated in human post-natal adrenal gland clusters (FDR<0.01).**FDRs were calculated with a Benjamini-Hochberg correction on one-sided Welch's *t*-tests (as detailed in Methods).

**Supplementary Data 4. Genes significantly up-regulated in mouse post-natal adrenal gland clusters (FDR<0.01).**FDRs were calculated with a Benjamini-Hochberg correction on one-sided Welch's *t*-tests (as detailed in Methods).

**Supplementary Data 5. Lists of cell populations in human post-natal adrenal glands sharing a significant specific gene signature with mouse post-natal-, human fetal adrenal glands [14], and developing (E13) mouse [6].** FDRs were calculated with a Benjamini-Hochberg correction on one-sided Fisher’s exact test (as detailed in Methods). * FDR<0.05, ** FDR<0.01.

**Supplementary Data 6. Genes significantly up-regulated in neuroblastoma clusters (FDR<0.01).**FDRs were calculated with a Benjamini-Hochberg correction on one-sided Welch's *t*-tests (as detailed in Methods).

**Supplementary Data 7. Transcriptional similarity between neuroblastoma and reference study cases.**Gene enrichment results for mesenchymal (MES) and adrenergic (ADR) signatures [16], and sympathetic noradrenergic (Group 1) and neural crest cell-like (Group 2) [15]. In addition, significant gene specific signature shared between cell populations in neuroblastoma with markers in GOSH neuroblastoma (10X-sequenced) cell clusters [13]. FDRs were calculated with a Benjamini-Hochberg correction on one-sided Fisher’s exact test (as detailed in Methods). * FDR<0.05, ** FDR<0.01.

**Supplementary Data 8. Lists of cell populations in neuroblastoma sharing a significant specific gene signature with mouse post-natal-, developing (E13) mouse [6], human post-natal-, and human fetal- [14] adrenal glands.** FDRs were calculated with a Benjamini-Hochberg correction on one-sided Fisher’s exact test (as detailed in Methods). * FDR<0.05, ** FDR<0.01.. + The original cluster annotations are currently debated and the included labels here correspond to those given by Kildisiute et al. [45], and Bedoya-Reina and Schlisio [46].

**Supplementary Data 9. Lists of genes in the specific signature of neuroblastoma cell populations significantly enriched in different risk- and survival groups, and significantly correlated with age at diagnosis.** FDRs were calculated with a Benjamini-Hochberg correction on one-sided Fisher’s exact test (as detailed in Methods). * FDR<0.05, ** FDR<0.01.

**Supplementary Data 10.** **Lists of biological processes (GO) enrichment for genes in the specific signature of neuroblastoma cell populations significantly enriched in different survival groups, and significantly correlated with age at diagnosis.** FDRs were calculated with a Benjamini-Hochberg correction on one-sided Fisher’s exact test (as detailed in Methods). * FDR<0.0*5*, ** FDR<0.01.

**Supplementary references**

[1] Bergman, J. et al. The human adrenal gland proteome defined by transcriptomics and antibody-based profiling. *Endocrinology* **158**, 239-251 (2017).

[2] Winkler, H. & Fischer-Colbrie, R. The chromogranins A and B: the first 25 years and future perspectives. *Neuroscience* **49**, 497-528 (1992).

[3] Carbone, E., Borges, R., Eiden, L., García, A. & Hernández-Cruz, A. Chromaffin Cells of the Adrenal Medulla: Physiology, Pharmacology, and Disease. 1443-1502 (2019).

[4] Pohorecky, L. & Wurtman, R. Adrenocortical control of epinephrine synthesis. *Pharmacological Reviews* **23**, 1-35 (1971).

[5] Albillos, A. & McIntosh, J. Human nicotinic receptors in chromaffin cells: characterization and pharmacology. *Pflügers Archiv - European Journal of Physiology* **470**, 21-27 (2018).

[6] Guérineau, N. Cholinergic and peptidergic neurotransmission in the adrenal medulla: A dynamic control of stimulus-secretion coupling. *IUBMB Life* **72**, 553-567 (2020).

[7] Hone, A. et al. Expression of α3β2β4 nicotinic acetylcholine receptors by rat adrenal chromaffin cells determined using novel conopeptide antagonists. *Journal of Neurochemistry* , (2020).

[8] Gahring, L., Myers, E., Palumbos, S. & Rogers, S. Nicotinic receptor Alpha7 expression during mouse adrenal gland development. *PLoS One* **9**, e103861 (2014).

[9] Bedoya-Reina, Oscar C. & Schlisio S. Chromaffin cells with sympathoblast signature: too similar to keep apart?. *Cancer Cell* **39**, 134-135 (2021).

[10] Takenobu, H. et al. CD133 suppresses neuroblastoma cell differentiation via signal pathway modification. *Oncogene* **30**, 97-105 (2011).

[11] Nguyen, B. et al. Cross-regulation between Notch and p63 in keratinocyte commitment to differentiation. *Genes & Development* **20**, 1028-1042 (2006).

[12] Schnurch, H. & Risau, W. Expression of tie-2, a member of a novel family of receptor tyrosine kinases, in the endothelial cell lineage. *Development* **119**, 957-968 (1993).

[13] François, M. et al. Sox18 induces development of the lymphatic vasculature in mice. *Nature* **456**, 643-647 (2008).

[14] Iljin, K. et al. A fluorescent Tie1 reporter allows monitoring of vascular development and endothelial cell isolation from transgenic mouse embryos. *FASEB Journal* **16**, 1764-1774 (2002).

[15] Gao, L. et al. Gene expression analyses reveal metabolic specifications in acute O2-sensing chemoreceptor cells. *Journal of Physiology* **595**, 6091-6120 (2017).

[16] Kamermans, A. et al. Setmelanotide, a novel, selective melanocortin receptor-4 agonist exerts anti-inflammatory actions in astrocytes and promotes an anti-inflammatory macrophage phenotype. *Frontiers in Immunology* **10**, 2312 (2019).

[17] Kim, W. et al. CD163 identifies perivascular macrophages in normal and viral encephalitic brains and potential precursors to perivascular macrophages in blood. *American Journal of Pathology* **168**, 822-834 (2006).

[18] Sintes, J., Romero, X., de Salort, J., Terhorst, C. & Engel, P. Mouse CD84 is a pan-leukocyte cell-surface molecule that modulates LPS-induced cytokine secretion by macrophages. *Journal of Leukocyte Biology* **88**, 687-697 (2010).

[19] Ryncarz, R. & Anasetti, C. Expression of CD86 on human marrow CD34+ cells identifies immunocompetent committed precursors of macrophages and dendritic cells. *Blood, The Journal of the American Society of Hematology* **91**, 3892-3900 (1998).

[20] Sehgal, A. et al. The role of CSF1R-dependent macrophages in control of the intestinal stem-cell niche. *Nature Communications* **9**, 1-17 (2018).

[21] Sen, A. & Ta, M. Altered Adhesion and Migration of Human Mesenchymal Stromal cells under febrile temperature Stress involves nf-κβ pathway. *Scientific Reports* **10**, 1-14 (2020).

[22] Picard, D. et al. Markers of survival and metastatic potential in childhood CNS primitive neuro-ectodermal brain tumours: an integrative genomic analysis. *Lancet Oncology* **13**, 838-848 (2012).

[23] Cheng, W., Kandel, J., Yamashiro, D., Canoll, P. & Anastassiou, D. Slug-based epithelial-mesenchymal transition gene signature is associated with prolonged time to recurrence in glioblastoma. *Nature Precedings* , 1-1 (2011).

[24] Agarwal, P. et al. Mesenchymal niche-specific expression of CXCL12 controls quiescence of treatment-resistant leukemia stem cells. *Cell Stem Cell* **24**, 769-784 (2019).

[25] Havis, E. et al. Transcriptomic analysis of mouse limb tendon cells during development. *Development* **141**, 3683-3696 (2014).

[26] Uezumi, A. et al. Identification and characterization of PDGFRα+ mesenchymal progenitors in human skeletal muscle. *Cell Death & Disease* **5**, e1186-e1186 (2014).

[27] Hosaka, K. et al. Pericyte-fibroblast transition promotes tumor growth and metastasis. *Proceedings of the National Academy of Sciences* **113**, E5618-E5627 (2016).

[28] van Groningen, T. et al. Neuroblastoma is composed of two super-enhancer-associated differentiation states. *Nature Genetics* **49**, 1261 (2017).

[29] Li, C. et al. Snail-induced claudin-11 prompts collective migration for tumour progression. *Nature Cell Biology* **21**, 251 (2019).

[30] Yang, Y., Wang, C. & Van Aelst, L. DOCK7 interacts with TACC3 to regulate interkinetic nuclear migration and cortical neurogenesis. *Nature Neuroscience* **15**, 1201-1210 (2012).

[31] Ryan, M., Tizard, R., VanDevanter, D. & Carter, W. Cloning of the LamA3 gene encoding the alpha 3 chain of the adhesive ligand epiligrin. Expression in wound repair. *Journal of Biological Chemistry* **269**, 22779-22787 (1994).

[32] Casazza, A. et al. Sema3E-Plexin D1 signaling drives human cancer cell invasiveness and metastatic spreading in mice. *Journal of Clinical Investigation* **120**, 2684-2698 (2010).

[33] Srivastava, A. et al. De novo dominant ASXL3 mutations alter H2A deubiquitination and transcription in Bainbridge-Ropers syndrome. *Human Molecular Genetics* **25**, 597-608 (2016).

[34] Khaled, W. et al. BCL11A is a triple-negative breast cancer gene with critical functions in stem and progenitor cells. *Nature Communications* **6**, 1-10 (2015).

[35] Britsch, S. et al. The ErbB2 and ErbB3 receptors and their ligand, neuregulin-1, are essential for development of the sympathetic nervous system. *Genes & Development* **12**, 1825-1836 (1998).

[36] Snippert, H. et al. Intestinal crypt homeostasis results from neutral competition between symmetrically dividing Lgr5 stem cells. *Cell* **143**, 134-144 (2010).

[37] Sanchez-Ortiz, E. et al. NF1 regulation of RAS/ERK signaling is required for appropriate granule neuron progenitor expansion and migration in cerebellar development. *Genes & Development* **28**, 2407-2420 (2014).

[38] Fiorino, A. et al. Retina-derived POU domain factor 1 coordinates expression of genes relevant to renal and neuronal development. *International Journal of Biochemistry & Cell Biology* **78**, 162-172 (2016).

[39] Kia, S. et al. RTTN mutations link primary cilia function to organization of the human cerebral cortex. *American Journal of Human Genetics* **91**, 533-540 (2012).

[40] Stolt, C., Lommes, P., Hillgärtner, S. & Wegner, M. The transcription factor Sox5 modulates Sox10 function during melanocyte development. *Nucleic Acids Research* **36**, 5427-5440 (2008).

[41] Hagiwara, N. Sox6, jack of all trades: a versatile regulatory protein in vertebrate development. *Developmental Dynamics* **240**, 1311-1321 (2011).

[42] Furlan, A. et al. Multipotent peripheral glial cells generate neuroendocrine cells of the adrenal medulla. *Science* **357**, eaal3753 (2017).

[43] Huber, K. et al. Development of chromaffin cells depends on MASH1 function. *Development* **129**, 4729-4738 (2002).

[44] Gil, D., Schamel, W., Montoya, M., Sánchez-Madrid, F. & Alarcón, B. Recruitment of Nck by CD3ϵ reveals a ligand-induced conformational change essential for T-cell receptor signaling and synapse formation. *Cell* **109**, 901-912 (2002).

[45] Wu, J. et al. An activating immunoreceptor complex formed by NKG2D and DAP10. *Science* **285**, 730-732 (1999).

[46] Natarajan, K. et al. An allosteric site in the T-cell receptor Cβ domain plays a critical signalling role. *Nature Communications* **8**, 1-14 (2017).

[47] Herold, S. et al. Recruitment of BRCA1 limits MYCN-driven accumulation of stalled RNA polymerase. *Nature* **567**, 545-549 (2019).[48] Rege, J. et al. Bone morphogenetic protein-4 (BMP4): a paracrine regulator of human adrenal C19 steroid synthesis. *Endocrinology* **156**, 2530-2540 (2015).

[49] Aragao-Santiago, L. et al. Mouse models of primary aldosteronism: from physiology to pathophysiology. *Endocrinology* **158**, 4129-4138 (2017).

[50] Rainey, W. Adrenal zonation: clues from 11β-hydroxylase and aldosterone synthase. *Molecular and Cellular Endocrinology* **151**, 151-160 (1999).

[51] Nishimoto, K. et al. Adrenocortical zonation in humans under normal and pathological conditions. *Journal of Clinical Endocrinology & Metabolism* **95**, 2296-2305 (2010).

[52] Peters, B. et al. StAR expression and the long-term aldosterone response to high-potassium diet in Wistar-Kyoto and spontaneously hypertensive rats. *American Journal of Physiology - Endocrinology and Metabolism* **292**, E16-E23 (2007).

[53] Nishimoto, K., Rainey, W., Bollag, W. & Seki, T. Lessons from the gene expression pattern of the rat zona glomerulosa. *Molecular and Cellular Endocrinology* **371**, 107-113 (2013).

[54] Allen, A., Zhuo, J. & Mendelsohn, F. Localization and function of angiotensin AT1 receptors. *American Journal of Hypertension* **13**, 31S-38S (2000).

[55] Aguilera, G. Role of angiotensin II receptor subtypes on the regulation of aldosterone secretion in the adrenal glomerulosa zone in the rat. *Molecular and Cellular Endocrinology* **90**, 53-60 (1992).

[56] Romero, D. et al. Disabled-2 is expressed in adrenal zona glomerulosa and is involved in aldosterone secretion. *Endocrinology* **148**, 2644-2652 (2007).

[57] Boulkroun, S. et al. Adrenal cortex remodeling and functional zona glomerulosa hyperplasia in primary aldosteronism. *Hypertension* **56**, 885-892 (2010).

[58] Wang, W., Yang, L., Suwa, T., Casson, P. & Hornsby, P. Differentially expressed genes in zona reticularis cells of the human adrenal cortex. *Molecular and Cellular Endocrinology* **173**, 127-134 (2001).

[59] Rainey, W. & Nakamura, Y. Regulation of the adrenal androgen biosynthesis. *Journal of Steroid Biochemistry and Molecular Biology* **108**, 281-286 (2008).

[60] Zah, E., Lin, M., Silva-Benedict, A., Jensen, M. & Chen, Y. T-cells expressing CD19/CD20 bispecific chimeric antigen receptors prevent antigen escape by malignant B cells. *Cancer Immunology Research* **4**, 498-508 (2016).

[61] Leadbetter, E. et al. Chromatin-IgG complexes activate B cells by dual engagement of IgM and Toll-like receptors. *Nature* **416**, 603-607 (2002).

[62] Kawabata, K., Ehata, S., Komuro, A., Takeuchi, K. & Miyazono, K. TGF-β-induced apoptosis of B-cell lymphoma Ramos cells through reduction of MS4A1/CD20. *Oncogene* **32**, 2096-2106 (2013).

[63] Vidal, V. et al. The adrenal capsule is a signaling center controlling cell renewal and zonation through Rspo3. *Genes & Development* **30**, 1389-1394 (2016).

[64] Oomori, Y., Habara, Y. & Kanno, T. Muscarinic and nicotinic receptor-mediated Ca 2+ dynamics in rat adrenal chromaffin cells during development. *Cell and Tissue Research* **294**, 109-123 (1998).

[65] Ya, J. et al. Sox4-deficiency syndrome in mice is an animal model for common trunk. *Circulation Research* **83**, 986-994 (1998).

[66] Clarke, R. et al. The expression of Sox17 identifies and regulates haemogenic endothelium. *Nature Cell Biology* **15**, 502-510 (2013).

[67] Scriba, L. et al. Cancer Stem Cells in Pheochromocytoma and Paraganglioma. *Frontiers in Endocrinology* **11**, 79 (2020).

[68] Dominguez-Soto, A. et al. Dendritic Cell-Specific ICAM-3-Grabbing Nonintegrin Expression on M2-Polarized and Tumor-Associated Macrophages Is Macrophage-CSF Dependent and Enhanced by Tumor-Derived IL-6 and IL-10. *Journal of Immunology* **186**, 2192-2200 (2011).

[69] Carr, M. et al. Mesenchymal precursor cells in adult nerves contribute to mammalian tissue repair and regeneration. *Cell Stem Cell* **24**, 240-256 (2019).

[70] Driscoll, T., Cosgrove, B., Heo, S., Shurden, Z. & Mauck, R. Cytoskeletal to nuclear strain transfer regulates YAP signaling in mesenchymal stem cells. *Biophysical journal* **108**, 2783-2793 (2015).

[71] Pinto, L. et al. AP2γ regulates basal progenitor fate in a region-and layer-specific manner in the developing cortex. *Nature Neuroscience* **12**, 1229 (2009).

[72] Rappl, G. et al. CD4+ CD7- leukemic T-cells from patients with Sezary syndrome are protected from galectin-1-triggered T-cell death. Leukemia 16, 840-845 (2002).

[73] Beilharz, E. et al. Neuronal activity induction of the stathmin-like gene RB3 in the rat hippocampus: possible role in neuronal plasticity. *Journal of Neuroscience* **18**, 9780-9789 (1998).

[74] Vervoort, S. et al. Global transcriptional analysis identifies a novel role for SOX4 in tumor-induced angiogenesis. *Elife* **7**, e27706 (2018).

[75] Qu, Y., Taylor, J., Bose, A. & Storkus, W. Therapeutic effectiveness of intratumorally delivered dendritic cells engineered to express the pro-inflammatory cytokine, interleukin (IL)-32. *Cancer Gene Therapy* **18**, 663-673 (2011).
