# Supplementary material for: Single-nuclei transcriptomes from human adrenal gland reveal distinct cellular identities of low and high-risk neuroblastoma tumors
Source: Nat Commun. 2021 Sep 7;12:5309. doi: 10.1038/s41467-021-24870-7 (PMC8423786; doi:10.1038/s41467-021-24870-7)
Supplement: Supplementary file 13 — Reporting Summary [file 41467_2021_24870_MOESM13_ESM.pdf]

nature research

Corresponding author(s): Bedoya-Reina O.C., Schlisio S.

Last updated by author(s): Jun 1, 2021

Reporting Summary

Nature Research wishes to improve the reproducibility of the work that we publish. This form provides structure for consistency and transparency in reporting. For further information on Nature Research policies, see our Editorial Policies and the Editorial Policy Checklist.

Statistics

For all statistical analyses, confirm that the following items are present in the figure legend, table legend, main text, or Methods section.

n/a	Confirmed	
☐	☒	The exact sample size (*n*) for each experimental group/condition, given as a discrete number and unit of measurement
☒	☐	A statement on whether measurements were taken from distinct samples or whether the same sample was measured repeatedly
☐	☒	The statistical test(s) used AND whether they are one- or two-sided<br>*Only common tests should be described solely by name; describe more complex techniques in the Methods section.*
☒	☐	A description of all covariates tested
☐	☒	A description of any assumptions or corrections, such as tests of normality and adjustment for multiple comparisons
☐	☒	A full description of the statistical parameters including central tendency (e.g. means) or other basic estimates (e.g. regression coefficient) AND variation (e.g. standard deviation) or associated estimates of uncertainty (e.g. confidence intervals)
☐	☒	For null hypothesis testing, the test statistic (e.g. *F*, *t*, *r*) with confidence intervals, effect sizes, degrees of freedom and *P* value noted<br>*Give P values as exact values whenever suitable.*
☒	☐	For Bayesian analysis, information on the choice of priors and Markov chain Monte Carlo settings
☐	☒	For hierarchical and complex designs, identification of the appropriate level for tests and full reporting of outcomes
☐	☒	Estimates of effect sizes (e.g. Cohen's *d*, Pearson's *r*), indicating how they were calculated

Our web collection on statistics for biologists contains articles on many of the points above.

Software and code

Policy information about availability of computer code

Data collection	Data collection: goldenpath mm10 mouse genome assembly available in the UCSC browser, goldenpath hg38 human genome assembly available in the UCSC browser, Genecode Human V28 comprehensive genome annotation, Genecode Mouse V18 comprehensive genome annotation, ENSEMBL GRCh38_p12 gene annotation, ENSEMBL GRCm38_p6 gene annotation, National Cancer Institute TARGET, dbGap Study Accession: phs000218.v16.p6, 498 SEQC dataset available in R2. E12 and E13 adrenal anlagen PAGODA rds from Furlan et al. 2017 [6], Reference gene sets for the GOSH cohort reported in Kildisiute et al. 2021 [13], Reference gene sets for human fetal adrenal gland from Dong et al. 2020 [14].
Data analysis	Reads processing and mapping: cutadapt 1.13, FastQC 0.11.5, star 2.5.3a, QoRTs 39cd1fc, rpy2 2.8.6, HTSeq 0.9.1, cellity 1.2.0, celloline 1808c31, GO.db 3.4.0, sgeostat 1.0-27.<br>PAGODA: cluster 2.0.7-1, lattice 0.20-38, scde 1.99.4, AnnotationDbi 1.40.0, org.Mm.eg.db 3.4.0, org.Hs.eg.db 3.4.0, Rtsne 0.15, Biobase 2.38.0, flexmix 2.3-13, IRanges 2.12.0, BiocGenerics 0.24.0, S4Vectors 0.16.0.<br>Expression differences analyses: scipy 0.18.1, h5py 2.10.0, numpy 1.12.0b1, pandas 0.22.0, scanpy 1.6.1.<br>Euclidean distances analysis: limma 3.40.6, pvclust 2.2-0, ape 5.3<br>Velocity, pseudotime, and entropy: palantir 0.2.6, scanpy 1.6.1, matplotlib 3.1.3, loompy 3.0.6, scvelo 0.2.2, velocyto 0.17.17.<br>Deconvolution: BiocGenerics 0.30.0, Biobase 2.44.0, BisqueRNA 1.0.4.<br>CNV analysis: inferCNV v1.4.0, CNAG v3.0, GDAS software.<br>Other analysis: Seurat 3.2.0, GeneSelector tool in R2, Kaplan Meier Scaner Pro tool in R2.

For manuscripts utilizing custom algorithms or software that are central to the research but not yet described in published literature, software must be made available to editors and reviewers. We strongly encourage code deposition in a community repository (e.g. GitHub). See the Nature Research guidelines for submitting code & software for further information.

Data

Policy information about availability of data

All manuscripts must include a data availability statement. This statement should provide the following information, where applicable:

- Accession codes, unique identifiers, or web links for publicly available datasets
- A list of figures that have associated raw data
- A description of any restrictions on data availability

Single cell/nuclei analysis results can be interactively browsed in the webpage "https://oxygen.mtc.ki.se/nc_nb_2021.html". Raw sequences for human adrenal gland are available in the Synapse ID project syn22301662 [https://www.synapse.org/#!Synapse:syn22302430]. Raw sequences for human adrenal gland sample 6657 are available with the Synapse IDs syn22302285 [https://www.synapse.org/#!Synapse:syn22302285] and syn25189163 [https://www.synapse.org/#!Synapse:syn25189163]. Raw sequences for human adrenal gland sample 6435 are available with the Synapse IDs syn22301836 [https://www.synapse.org/#!Synapse:syn22301836]. Raw sequences for human adrenal gland sample 16-D are available with the Synapse IDs syn22301667 [https://www.synapse.org/#!Synapse:syn22301667]. Raw sequences for neuroblastoma are available in the Synapse ID project syn22302605 [https://www.synapse.org/#!Synapse:syn22310692]. Raw sequences for neuroblastoma sample K87 are available in the Synapse ID syn22307346 [https://www.synapse.org/#!Synapse:syn22307346]. Raw sequences for neuroblastoma sample K6 are available in the Synapse ID syn22306928 [https://www.synapse.org/#!Synapse:syn22306928]. Raw sequences for neuroblastoma sample K55 are available in the Synapse ID syn22306349 [https://www.synapse.org/#!Synapse:syn22306349]. Raw sequences for neuroblastoma sample K47 are available in the Synapse ID syn22305928 [https://www.synapse.org/#!Synapse:syn22305928]. Raw sequences for neuroblastoma sample K40 are available in the Synapse ID syn22305408 [https://www.synapse.org/#!Synapse:syn22305408]. Raw sequences for neuroblastoma sample K3 are available in the Synapse ID syn22304935 [https://www.synapse.org/#!Synapse:syn22304935]. Raw sequences for neuroblastoma sample K2 are available in the Synapse ID syn22304482 [https://www.synapse.org/#!Synapse:syn22304482]. Raw sequences for neuroblastoma sample K14 are available in the Synapse ID syn22304038 [https://www.synapse.org/#!Synapse:syn22304038]. Raw sequences for neuroblastoma sample K10 are available in the Synapse ID syn22303649 [https://www.synapse.org/#!Synapse:syn22303649]. Raw sequences for neuroblastoma sample 23 are available in the Synapse ID syn22303256 [https://www.synapse.org/#!Synapse:syn22303256]. Raw sequences for neuroblastoma sample 19 are available in the Synapse ID syn22302820 [https://www.synapse.org/#!Synapse:syn22302820]. Raw sequences for mouse adrenal gland are available in the Synapse ID project syn22308005 [https://www.synapse.org/#!Synapse:syn22310690]. Raw sequences for mouse adrenal gland sample 6801 are available in the Synapse ID syn22308008 [https://www.synapse.org/#!Synapse:syn22308008]. Raw sequences for mouse adrenal gland sample 6802 are available in the Synapse ID syn22308616 [https://www.synapse.org/#!Synapse:syn22308616]. Raw sequences for mouse adrenal gland sample 3431 are available in the Synapse ID syn22309301 [https://www.synapse.org/#!Synapse:syn22309301]. Raw sequences for mouse adrenal gland sample 3432 are available in the Synapse ID syn22310231 [https://www.synapse.org/#!Synapse:syn22310231]. Raw sequences for mouse adrenal gland sample I1 are available in the Synapse ID syn22309843 [https://www.synapse.org/#!Synapse:syn22309843]. RNA-sequences from neuroblastoma available at: National Cancer Institute TARGET, dbGap Study Accession phs000218.v16.p6. The remaining data are available within the Article, Supplementary Information or available from the authors upon request. Data analysis pipeline is available in https://github.com/oscarcbr/nc_nb_2021.

Field-specific reporting

Please select the one below that is the best fit for your research. If you are not sure, read the appropriate sections before making your selection.

[x] Life sciences   [ ] Behavioural & social sciences   [ ] Ecological, evolutionary & environmental sciences

For a reference copy of the document with all sections, see nature.com/documents/nr-reporting-summary-flat.pdf

Life sciences study design

All studies must disclose on these points even when the disclosure is negative.

Sample size	No statistical tests or analyses were used to predetermine sample size. The patient material was limited, and we followed standards in the field. A total of 5 mouse adrenal glands, 3 human adrenal glands and 11 neuroblastomas were included in this study to analyze at least three samples for each adrenal gland postnatal time point or neuroblastoma subgroup.
Data exclusions	none
Replication	Not possible due to sample availability limitations
Randomization	No randomization was performed for this study. Groups were defined by neuroblastoma subtype or adrenal gland postnatal time point.
Blinding	Blinding was not relevant in this study. Tumors and normal tissues were not grouped into experimental groups, so measurement and data did not require subjective judgement from the investigators.

Reporting for specific materials, systems and methods

We require information from authors about some types of materials, experimental systems and methods used in many studies. Here, indicate whether each material, system or method listed is relevant to your study. If you are not sure if a list item applies to your research, read the appropriate section before selecting a response.

Materials & experimental systems

n/a	Involved in the study	
x		Antibodies
x		Eukaryotic cell lines
x		Palaeontology and archaeology
	x	Animals and other organisms
	x	Human research participants
x		Clinical data
x		Dual use research of concern

Methods

n/a	Involved in the study	
x		ChIP-seq
x		Flow cytometry
x		MRI-based neuroimaging

Animals and other organisms

Policy information about studies involving animals; ARRIVE guidelines recommended for reporting animal research

Laboratory animals	C57BL/6 mice were kept in rooms with controlled 12-h light/dark cycles, temperature, and humidity with food and water provided. The mice were housed at a maximum of four males per cage and four females per cage. Animal care procedures were in accordance with the guidelines set by the European Community Council Directives (86/609/EEC). Required animal permissions were obtained from the local ethical committee.<br>manuscript on page 27-28 and details available in Supplementary Data 1
Wild animals	No wild animals were used in the study.
Field-collected samples	No field collected samples were used in the study.
Ethics oversight	All animal experiments were performed according to Swedish guidelines and regulations, and the ethical permit 7694-2017 was granted by Stockholms Norra djurfšrsšksetiska nämnd, Sweden.

Note that full information on the approval of the study protocol must also be provided in the manuscript.

Human research participants

Policy information about studies involving human research participants

Population characteristics	Details available in Supplementary Data 1
Recruitment	Deep frozen patients tumors samples were selected for this study based on availability of material. No self-selection bias or other biases was present and thus did not impact results.
Ethics oversight	Human samples were collected under the ethical permits from Stockholm Regional Ethical Review Board and the Karolinska University Hospital Research Ethics Committee (2009/1369-31/1 and 03-736) and KI 2007/069 and KI 2001/136 , issued to Professor's P. Kogner and C. Larrson for neuroblastoma and adrenal gland samples, respectively. All samples were obtained following an informed patient consent. Additional post-mortem human adrenal glands for staining's were obtained from the NIH Neurobiobank (University of Maryland, Baltimore, MD) under the same ethical permit from Stockholm Regional Ethical Review Board and the Karolinska University Hospital Research Ethics Committee.

Note that full information on the approval of the study protocol must also be provided in the manuscript.
